# Generation and Next-Generation Sequencing-Based Characterization of a Large Human Combinatorial Antibody Library

**DOI:** 10.3390/ijms24066011

**Published:** 2023-03-22

**Authors:** Hye Lim Choi, Ha Rim Yang, Ha Gyeong Shin, Kyusang Hwang, Ji Woong Kim, Ji Hyun Lee, Taehoon Ryu, Yushin Jung, Sukmook Lee

**Affiliations:** 1 Department of Biopharmaceutical Chemistry, Kookmin University, Seoul 02707, Republic of Korea; 2 Department of Chemistry, Kookmin University, Seoul 02707, Republic of Korea; 3 ATG Lifetech Inc., Seoul 08507, Republic of Korea; 4 Department of Applied Chemistry, Kookmin University, Seoul 02707, Republic of Korea; 5 Antibody Research Institute, Kookmin University, Seoul 02707, Republic of Korea

**Keywords:** antibody library, human monoclonal antibody, next-generation sequencing, phage display, somatic hypermutation

## Abstract

Antibody phage display is a key technology for the discovery and development of target-specific monoclonal antibodies (mAbs) for use in research, diagnostics, and therapy. The construction of a high-quality antibody library, with larger and more diverse antibody repertoires, is essential for the successful development of phage display-derived mAbs. In this study, a large human combinatorial single-chain variable fragment library (1.5 × 10^11^ colonies) was constructed from Epstein–Barr virus-infected human peripheral blood mononuclear cells stimulated with a combination of two of the activators of human B cells, the Toll-like receptor 7/8 agonist R848 and interleukin-2. Next-generation sequencing analysis with approximately 1.9 × 10^6^ and 2.7 × 10^6^ full-length sequences of heavy chain variable (VH) and κ light chain variable (Vκ) domains, respectively, revealed that the library consists of unique VH (approximately 94%) and Vκ (approximately 91%) sequences with greater diversity than germline sequences. Lastly, multiple unique mAbs with high affinity and broad cross-species reactivity could be isolated from the library against two therapeutically relevant target antigens, validating the library quality. These findings suggest that the novel antibody library we have developed may be useful for the rapid development of target-specific phage display-derived recombinant human mAbs for use in therapeutic and diagnostic applications.

## 1. Introduction

Monoclonal antibody (mAb) technology is useful, not only for elucidating the pathological mechanism in disease progression, but also for the diagnosis and treatment of a variety of diseases, including cancers, neurological and inflammatory disorders, and infectious diseases [1,2]. Traditionally, immunization and hybridoma technology, which involves the production of a hybrid cell and which is formed via the fusion between short-lived antibody-producing B cells and those of an immortal myeloma, have been used successively to identify and overproduce target-specific mAbs [3,4]. Through this technology, OKT3, the first United States Food and Drug Administration-approved therapeutic antibody against CD3 epsilon, was generated to improve graft survival in patients exhibiting renal allograft rejection [5]. However, these processes are time-consuming and labor-intensive and result in high immunogenicity risk for therapeutic applications [6]. Recent advances in recombinant DNA technology have enabled antibody humanization, the generation of recombinant human antibody libraries, phage display antibody selection, and the overproduction of phage display-derived mAbs [7,8]. Currently, antibody phage display is the universal antibody selection technology used to rapidly develop target-specific mAbs from an established antibody library for a wide range of academic and industrial applications [9,10].

The construction of a high-quality antibody library depends on the diversity of the antibody repertoire [11]. The antigen binding site of an antibody is composed of the variable domains of the heavy (VH) and light (VL) chains of its antigen binding fragment. Each VH and VL domain of an antibody contain three complementarity-determining regions (CDRs), which are the main regions engaged in antigen binding, and four framework regions. CDR1 and CDR2 are encoded in each V germline gene segment, whereas the heavy chain CDR3 (HCDR3) is formed by V(D)J recombination and the light chain CDR3 (LCDR3) by VJ recombination [12]. V(D)J recombination and somatic hypermutation (SHM) are the primary mechanisms responsible for the diversification of the human antibody repertoire [13]. These allow for rapid humoral immune responses to a wide range of antigenic challenges [14]. In B cell receptor engagement, the characteristic feature of SHM is the upregulation of the expression of activation-induced cytidine deaminase (AID), which deaminates deoxycytidine residues in single-stranded DNA to deoxyuridines [15,16]. This results in increased random point mutations within the immunoglobulin (Ig) germline genes and produces affinity-matured Igs with high affinity [17]. On the other hand, the process of V(D)J recombination is the generation of high-diversity antibody repertoires through the random recombination of V(D)J or VJ genes [18]. This process is performed by the use of recombination-activating genes such as RAG1 and RAG2, known as V(D)J recombinases [19].

Generating mAbs is a labor-intensive and time-consuming process that requires the immunization of the host individuals [20]. The transfer of the humoral immune response into in vitro settings enables the shortening of this process and circumvents the necessity of in vivo immunization [21]. To date, multiple in vitro immunization methods have been studied for the efficient production of human mAbs. Several reports have shown that in vitro stimulation of human peripheral blood mononuclear cells (hPBMCs), using various cytokines, antigens, or adjuvants, can elicit the expansion of B-cells to produce mAbs and enhance the diversity of the antibody repertoire [22,23,24]. More recently, R848 (Resiquimod), an imidazoquinoline, has been identified as a dual Toll-like receptor 7 (TLR7) and TLR8 synthetic agonist with potent immunostimulatory activity. Some studies also report that R848 plays a key role in B cell proliferation and differentiation into Ig-secreting cells and in the upregulation of AID, a marker protein used to initiate SHM [25,26,27,28]. Furthermore, Pinna et al. revealed that the combinatorial treatment of R848 and interleukin-2 (IL-2) is an efficient and synergistic method for the in vitro stimulation of resting B cells into Ig-secreting cells [29].

Next-generation sequencing (NGS) is a powerful tool for the massive parallel sequencing of a whole genome or of specific regions of interest in the genome [30]. Recent advances in NGS technology enable rapid identification of large-scale gene expression patterns and genetic variations at a relatively low cost [31]. Sanger sequencing is a first-generation DNA sequencing method based on electrophoresis and the random incorporation of chain-terminating dideoxynucleotides [32]. Despite initially being used widely, Sanger sequencing has several limitations, including labor intensiveness, high cost, and limited throughput [33]. In contrast, NGS can analyze millions to billions of DNA fragments simultaneously, thereby overcoming the limitations of Sanger sequencing [34]. This makes NGS suitable for use in rapid and efficient DNA sequencing. Thus, NGS can be widely used for multiple applications, including for the high-throughput characterization required for the quality assessment of antibody libraries [35].

In this study, a large human combinatorial single-chain variable fragment (scFv) library was generated from Epstein–Barr virus (EBV)-infected hPBMCs, stimulated with a combination of R848 and IL-2 to mimic antibody repertoire diversification in human B cell activation. The diversity and quality assessments of the library were verified by isolating and analyzing phage display-derived mAbs from the library against two therapeutically relevant antigens, as well as by high-throughput NGS analysis. These findings provide a useful tool for the rapid development of target-specific and phage display-derived mAbs from an established antibody library and offer fundamental insights into the futuristic strategy of functional paratope diversification.

## 2. Results

### 2.1. Construction of a Novel Human Combinatorial Antibody Library from Activated B-Cells

**Figure 1:**
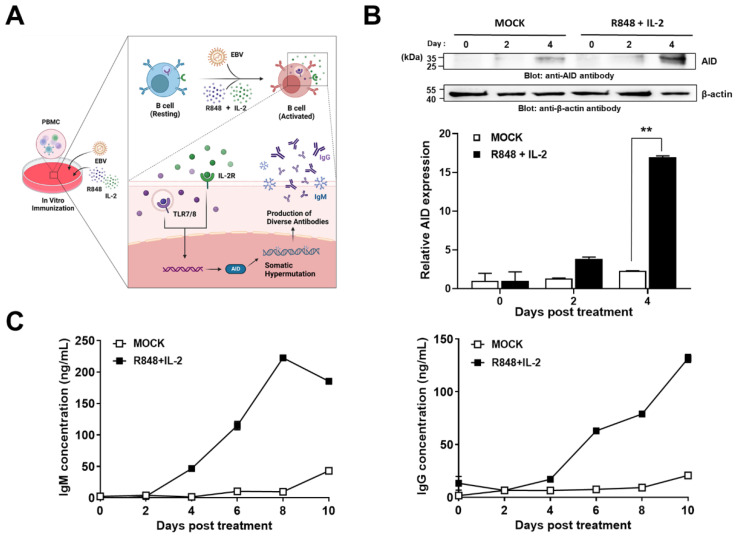
In vitro immunization and biochemical characterization of human peripheral blood mononuclear cells (hPBMCs) stimulated with a combination of R848 and interleukin-2 (IL-2). (**A**) Schematic representation showing overall experimental procedures and key characteristic mechanisms in B cell activation. hPBMCs were cultured in the presence or absence of a combination of R848 and IL-2 for the indicated time. (**B**) The prepared cell extracts were subjected to immunoblot analysis to check the protein expression of activation-induced cytidine deaminase. After the quantitation of band intensity, the protein expression was represented on a bar graph. (**C**) Immunoglobulin M or immunoglobulin G production from the culture supernatants of the cultured hPBMCs, obtained every two days after the treatment, was analyzed using enzyme-linked immunosorbent assay. All values represent the mean ± standard error of the mean from duplicate measurements of one of two independent experiments. Data were statistically analyzed with two-tailed Student’s *t* tests (** *p* < 0.01).

To generate a large human antibody library with high diversity, it is essential to acquire activated B cell populations that possess more diverse antibody repertoires than naïve B cells. For this purpose, we attempted to establish an in vitro immunization setting. In detail, hPBMCs which had been infected with EBV to obtain the immortalized B-cells were stimulated with or without a combination of R848 and recombinant human IL-2 (rhIL-2), both well-known potent activators of B-cells (Figure 1A). Then, the cell extracts were subjected to immunoblot analysis to investigate the protein expression of AID, which plays a key role in antibody repertoire diversification. We found that AID was highly upregulated (seven times higher than non-treated groups) in agonist-treated hPBMCs on day 4 (Figure 1B).

To investigate the agonist-induced antibody production from the activated B cells in the established in vitro condition, we performed enzyme-linked immunosorbent assay (ELISA) and measured the amount of IgG or IgM secreted from hPBMCs which had been stimulated with or without a combination of R848 and rhIL-2, respectively. The production of IgM and IgG distinctly increased from 4 days after the treatment in a time-dependent manner compared to control groups. Further, on day 10, the production levels of IgM and IgG significantly increased 4.3-fold and 6.3-fold, respectively, compared to the control group (Figure 1C).

The total RNA of the hPBMCs on day 10 after agonist treatment was isolated and reverse-transcribed into complementary DNA (cDNA). Each VH and Vκ gene was individually amplified by polymerase chain reaction (PCR) from the cDNA using the gene-specific primers for all VH and Vκ germline genes (Supplementary Table S1). Subsequently, amplified VH and Vκ repertoires were randomly associated in a scFv VH-linker-VL architecture. Then, the scFvs were cloned into the SfiI sites of the pComb3XSS phagemid DNA. Finally, the resulting scFv library was introduced into *Escherichia coli* (*E. coli)* ER2738 by electroporation, and a novel human antibody library size was achieved with 1.5 × 10^11^ clones.

### 2.2. NGS of the Constructed Human scFv Antibody Library

**Figure 2:**
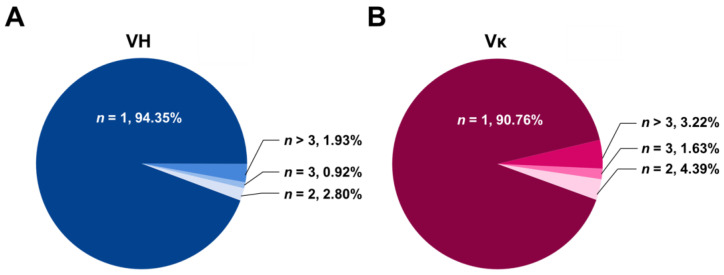
Redundancy of the variable domain sequences of the constructed library. The number of replicates (*n*) is expressed as a percentage of each heavy chain variable (VH) (**A**) and κ light chain variable (Vκ) (**B**) domain sequence out of the total analyzed sequences. Approximately 94% of VH and 91% of Vκ sequences were found to be unique sequences as shown here (*n* = 1).

To analyze the diversity of VH and Vκ repertoires in the human scFv antibody library, the phagemid DNA sequences encoding the scFv clones were isolated from the *E. coli* transformants, and the VH and Vκ amplicon libraries were generated on the basis of the PCR technique in order to conduct NGS. Initially, two (forward and reverse) sequencing runs yielded 2,516,495 VH and 3,148,981 Vκ reads. Multiple processing steps and filterings of NGS data were performed to eliminate the reads containing sequencing artifacts, stop codons, and/or out-of-sequence frames. Then, we obtained the selected 1,865,467 VH and 2,723,431 Vκ reads, respectively. Next, we attempted to identify the redundancy of each VH and Vκ domain sequence in the reads. We discovered that approximately 94% of VH and 91% of Vκ sequences occurred only once (Figure 2), indicating that the constructed library is mainly composed of unique antibody clones.

### 2.3. Germline Distribution Analysis of the Antibody Library Clones

**Figure 3:**
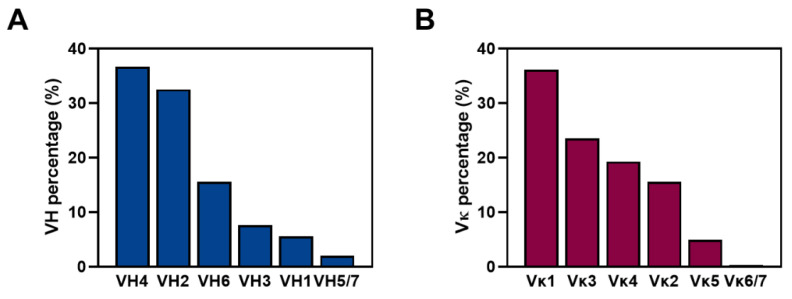
Abundance of each variable domain subfamily observed in the constructed library. Following the germline classification of the antibody sequences in the library, each heavy chain variable (blue) (**A**) and κ light chain variable (red) (**B**) domain subfamily is depicted using percentage bar graphs in descending order.

For the germline classification of the antibody sequences in the library, the 1,865,467 VH and 2,723,431 Vκ sequences were analyzed and classified by the international ImMunoGeneTics (IMGT) human antibody database using IgBLAST analysis tool. Each sequence was categorized into one of the 66 VH or 50 Vκ germline gene segments. Further, the abundance of each variable domain family and gene segment observed in the library was analyzed and represented as a percentage, respectively. The most abundant VH subfamilies were VH4 (36.8%) > VH2 (32.5%) > VH6 (15.6%) > VH3 (7.6%) > VH1 (5.5%) > VH5 (1.5%) > VH7 (0.5%) (Figure 3A), whereas the most abundant Vκ subfamilies were Vκ1 (36.2%) > Vκ3 (23.6%) > Vκ4 (19.3%) > Vκ2 (15.6%) > Vκ5 (5.0%) > Vκ6 (0.3%) > Vκ7 (0.0045%) (Figure 3B). Furthermore, the main VH gene segments in each identified subfamily were VH1-69 (1.9%), VH1-2 (1.1%), VH2-5 (21.2%), VH2-26 (6.4%), VH2-70 (4.9%), VH3-23 (1.2%), VH3-30 (1.1%), VH4-34 (18.9%), VH4-39 (6.0%), VH4-59 (3.5%), and VH4-38-2 (2.2%), whereas the identified Vκ gene segments were Vκ1-39 (7.8%), Vκ1-33 (5.8%), Vκ1-9 (5.0%), Vκ1-5 (2.9%), Vκ1-12 (2.4%), Vκ1-13 (2.1%), Vκ2-28 (5.3%), Vκ2-30 (5.2%), Vκ2D-29 (2.8%), Vκ3-11 (4.0%), Vκ4-1 (19.3%), and Vκ5-2 (5.0%).

**Figure 4:**
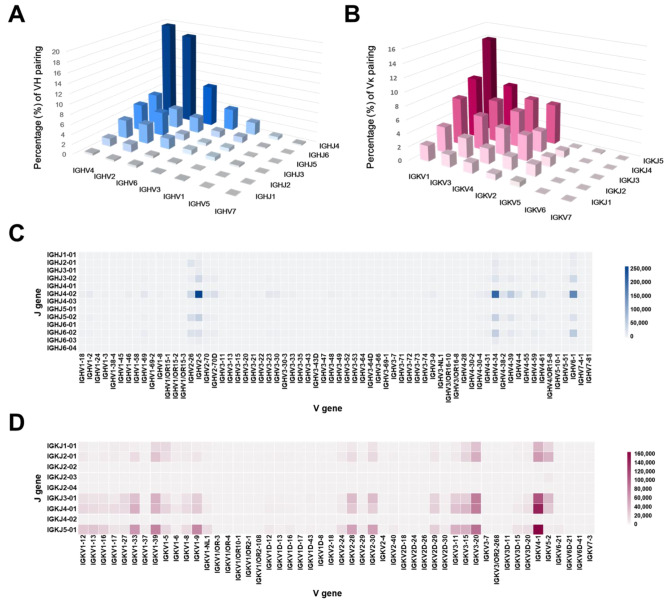
Pairing and frequencies of germline V and J gene families and segments in each heavy chain variable (VH, blue) and κ light chain variable (Vκ, red) domain of the constructed library. The pairs of germline V and J gene families in VH (**A**) or Vκ (**B**) are expressed as percentage bar graphs. The pairing frequencies of 66 IGHV and 13 IGHJ gene segments in VH (**C**) or 50 IGκV and 9 IGκJ in Vκ (**D**) are illustrated as two-dimensional heatmaps.

To investigate the diversity of the antibody gene repertoire in the constructed library, the pairing and frequencies of germline V and J gene families in each VH or Vκ in the library were also analyzed. Each sequence was categorized into one of the pairs of the seven IGHV and six IGHJ families in VH or seven IGκV and five IGκJ families in Vκ. In detail, the most frequently found pairs in the VH family, in descending order, were IGHV4_IGVJ4 (20.0%), IGHV2_IGVJ4 (18.1%), IGHV6_IGVJ4 (8.2%), IGHV4_IGVJ6 (6%), IGHV4_IGVJ5 (5.3%), IGHV2_IGVJ5 (4.7%), IGHV3_IGVJ4 (4.3%), IGHV2_IGVJ6 (4.0%), IGHV2_IGVJ3 (3.9%), IGHV4_IGVJ3 (3.7%), and IGHV6_IGVJ6 (3.1%) (Figure 4A), whereas those in the Vκ family were IGκV1_IGκJ5 (14.1%), IGκV1_IGκJ4 (9.0%), IGκV3_IGκJ5 (7.5%), IGκV1_IGκJ3 (7.0%), IGκV3_IGκJ4 (6.2%), IGκV3_IGκJ5 (6.1%), IGκV1_IGκJ5 (6.0%), IGκV3_IGκJ4 (5.3%), IGκV1_IGκJ3 (5.1%), IGκV3_IGκJ3 (4.0%), IGκV1_IGκJ2 (3.8%), IGκV1_IGκJ3 (3.8%), and IGκV1_IGκJ4 (3.1%) (Figure 4B). To further confirm the overall tendency of the VJ recombination in the constructed library, we generated two-dimensional heatmaps to show the pairs and frequencies of individual 66 IGHV and 13 IGHJ or 50 IGκV and 9 IGκJ gene segments in each VH or Vκ families (Figure 4C,D). As a result, we found that a variety of VJ gene combinations in both VH and Vκ families were evenly distributed in the library. Moreover, the major pairs of IGHV and IGHJ genes in VH, in descending order, were IGHV2-5_IGHJ4-02 (13.3%), IGHV4-34_IGHJ4-02 (10.7%), IGHV6-1_IGHJ4-02 (8.1%), IGHV4-39_IGHJ4-02 (3.1%), and IGHV6-1_IGHJ6-02 (3.0%), whereas those of IGκV and IGκJ in Vκ were IGκV4-1_IGκJ5-01 (6.1%), IGκV4-1_IGκJ4-01 (5.3%), IGκV4-1_IGκJ3-01 (4.0%), IGκV3-20_IGκJ5-01 (3.3%), and IGκV4-1_IGκJ4-01 (3.0%) (Supplementary Table S2).

### 2.4. Diversity Assessment of the Length and Amino Acid Composition of CDRs

**Figure 5:**
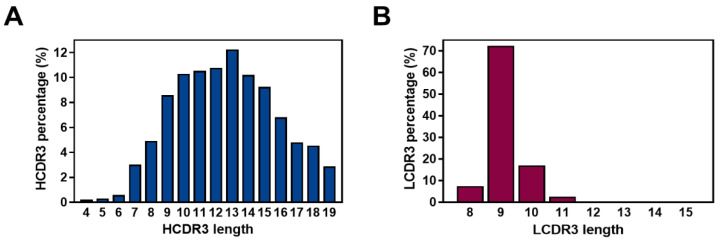
Length diversity of heavy and light chain complementarity-determining region 3 (CDR3) in the constructed library. Frequencies of heavy chain CDR3 (HCDR3, blue) (**A**) and light chain CDR3 (LCDR3, red) (**B**) with diverse lengths are expressed as percentage bar graphs. The HCDR3 and LCDR3 length ranges were 4–19 and 8–15 amino acids, respectively.

Analysis of the 1,865,467 VH sequences revealed that HCDR3 lengths ranged from 4 to 19 amino acids, with 13 amino acids being the most frequently occurring length (Figure 5A). For VH, the amino acid usages at each position in HCDR1 (Kabat number 31–35), HCDR2 (Kabat number 50–65), or HCDR3 (Kabat number 95–102) in the library were determined and are below displayed in each stacked bar (Supplementary Table S3). We found that, in the HCDR3 amino acids in the library, cysteine residues which may involve disulfide bond formation were present at a relatively lower level (1.0%), whereas phenylalanine (14.0%), tyrosine (13.7%), glycine (11.6%), and serine (7.0%) were favored (Supplementary Table S4) [36].

**Figure 6:**
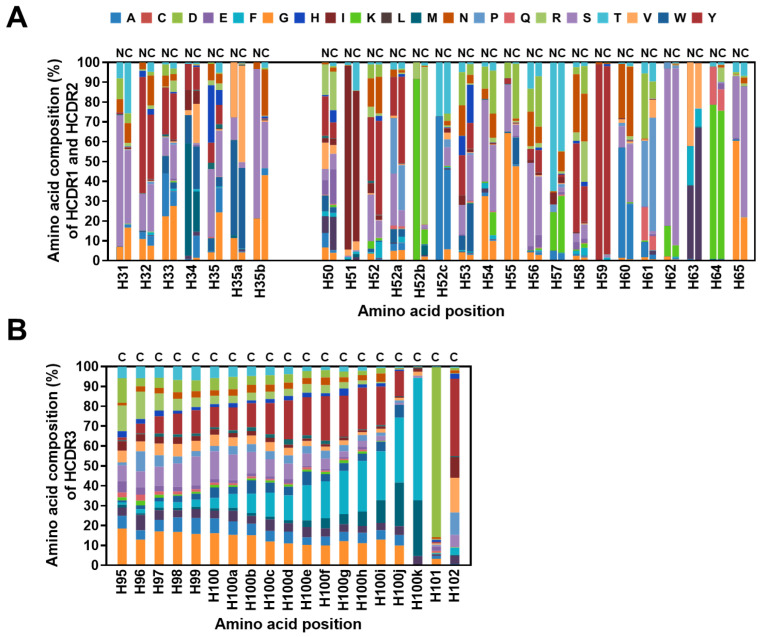
Diversity of the amino acid composition in the heavy chain complementarity-determining regions (HCDRs) of the constructed library. (**A**) The amino acid compositions at each position within HCDR1 and HCDR2 of natural human antibodies (N) and in the constructed library (C) were comparatively analyzed. The frequencies of amino acids occurring in each position of HCDR1 and HCDR2 (**A**) were compared with those in the reference sequences and are shown as percentages and represented in each stacked bar with designated colors. (**B**) Similarly, the amino acid composition of HCDR3 was analyzed and expressed as a percentage and represented in each stacked bar with designated colors. The *x* axis shows the amino acid position based on Kabat numbering.

Then, the position-specific amino acid usages within the library were compared with those in the corresponding V germline gene segments as reference genes obtained from the IMGT database. The data showed that the amino acid compositions of the HCDR1 and HCDR2 in the library appear to have distribution patterns which are distinct from those of the germline gene segments, indicating the increased diversity of CDR sequences in the library (Figure 6A,B).

**Figure 7:**
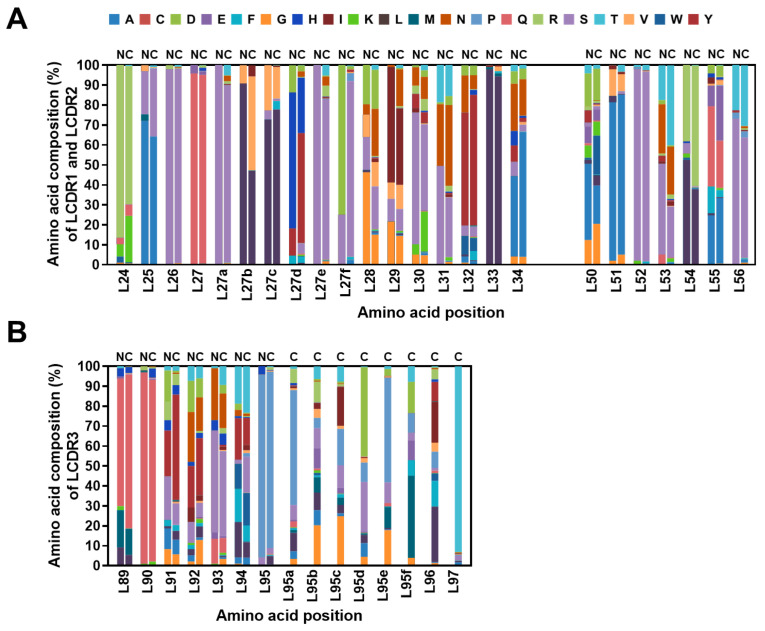
Diversity of the amino acid composition in the light chain complementarity-determining regions (LCDRs) of the constructed library. (**A**) The amino acid compositions at each position within LCDR1 and LCDR2 of natural human antibodies (N) were analyzed in comparison to those of the constructed library (C). The frequencies of amino acid occurrence in each position of LCDR1 and LCDR2 were compared with the reference sequences. (**B**) The amino acid incidence in LCDR3 was expressed as a percentage and represented in each stacked bar with designated colors. The *x* axis shows the amino acid position based on Kabat numbering.

An analysis of the 2,723,431 Vκ sequences revealed that LCDR3 length range to be 8–15 amino acids, with a length of nine amino acids being predominant (Figure 5B). We determined the amino acid composition at each position in the LCDR1 (Kabat number 24–34), LCDR2 (Kabat number 50–56), or LCDR3 (Kabat number 89–97) (Supplementary Table S5). The result showed that throughout LCDR3 amino acids in the library, proline (16.8%), glutamine (12.2%), and threonine (10.7%) are favored (Supplementary Table S6), whereas the incidence of cysteine residue is shown to be negligible (0.1%). By comparing amino acid usages within the sequences of the library with those of the germline gene segments, we also observed that, despite some LCDR amino acids in the library having a pattern similar to those in germline reference sequences, overall, the position-specific amino acid usage in the library appears to be distinct from that in germline gene segments, which also suggests the increased diversity in amino acid usage across the LCDRs (Figure 7A,B).

**Figure 8:**
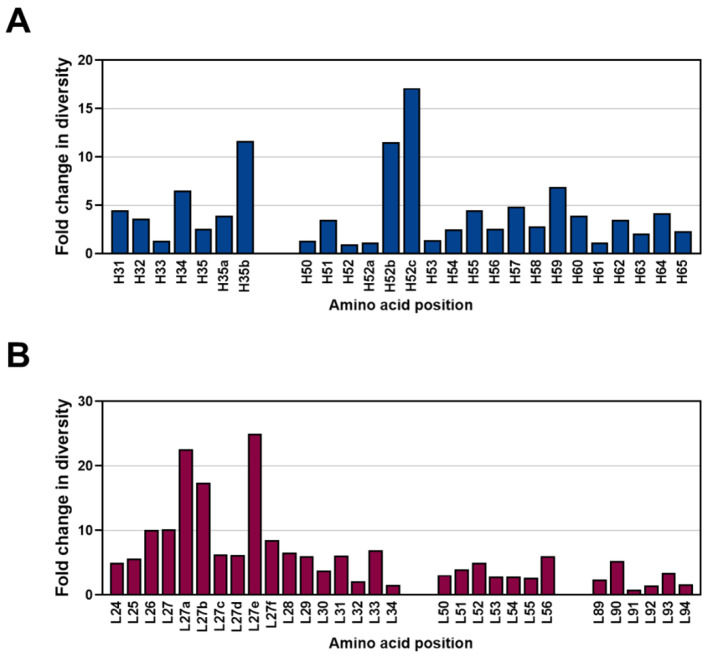
Analysis of the diversity in heavy chain complementarity-determining regions (HCDRs) and light chain complementarity-determining regions (LCDRs) in the constructed library. The HCDR1 and HCDR2 of heavy chain variable domain (**A**) or LCDR1, LCDR2 and LCDR3 of light chain variable domain (**B**) are shown in blue and red colors, respectively. Individual diversity equals the total number of different residues at each position, divided by the relative frequency of the most common residue at that position. The *x* axis shows the amino acid position based on Kabat numbering.

To quantitate the sequence diversity of HCDRs and LCDRs in the constructed library, we analyzed the individual diversities that are equal to the total number of different residues at each position, which were divided by the relative frequency of the most common residue at that position in the library or in the germline reference sequences from the IMGT database. Then, the diversity ratio between the library and the reference sequences was calculated and visualized. The data revealed that the diversity is greatest at H52c, followed by H35b, H52b, H59, and H34 in HCDR1 and HCDR2 (Figure 8A), whereas the diversity at L27e is greatest, followed by L27a or L27b L27 and L26 in LCDR1, LCDR2, and LCDR3 (Figure 8B). We also found that the constructed library had approximately 4.3 times or 6.4 times greater diversity than the natural antibody sequences in each VH or Vκ on average, respectively.

### 2.5. Frequencies of SHMs in CDRs

**Figure 9:**
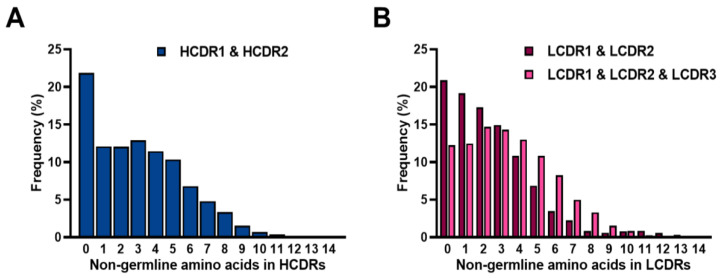
The frequency of non-germline amino acids in heavy chain complementarity-determining regions (HCDRs) or light chain complementarity-determining regions (LCDRs) in the constructed library. The numbers of non-germline amino acid residues found in HCDR1 and HCDR2 (**A**), LCDR1 and LCDR2, or LCDR1 and LCDR2 and LCDR3 (**B**) of the analyzed heavy chain variable domain (blue) and κ light chain variable domain (red) sequences were expressed as percentage bar graphs. The *x* axis shows the number of non-germline amino acids in CDRs.

To analyze the frequency of SHM in the constructed library, we first applied more stringent filtering to determine the correct alignment of the CDR regions. Based on these alignments, we compared the sequences of the tested samples with those of the reference germline sequences. For this purpose, the 1,299,842 VH and 1,714,662 Vκ sequences were compared with the most closely aligned germline VH or Vκ gene segments in IMGT human antibody database using the IgBLAST analysis tool. More specifically, the frequencies of non-germline amino acids observed in the constructed library were calculated by comparing the CDR amino acid sequences of the obtained VH and Vκ with those of the germline reference sequences. We found that 22% of HCDR1 and HCDR2 out of the 1,299,842 VH sequences had no SHM, whereas 66% of them had between 1–6 mutations, and the remaining 12% had more than six mutations. HCDR1 and HCDR2 had five amino acids on average (Figure 9A). In the case of LCDR1 and LCDR2 in Vκ, 21% of LCDR sequences had no mutation, while 73% of them had between 1–6 mutations. The remaining 6% had more than six mutations. Three amino acids in LCDR1 and LCDR2 on average were mutated. In cases of LCDR1, LCDR2 and LCDR3 in Vκ, 12% had no mutation, 74% had between 1–6 mutations, and the remaining 14% had more than six mutations, while average of five amino acids were mutated (Figure 9B).

**Figure 10:**
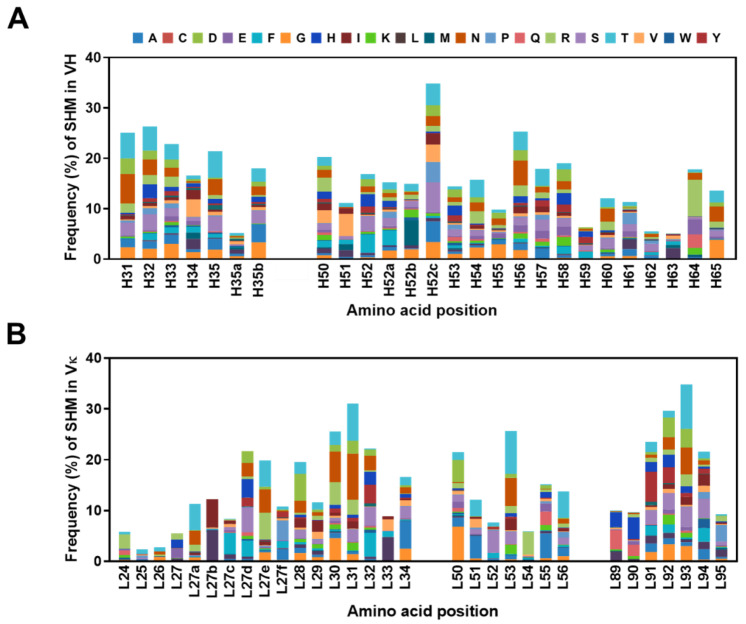
Position-specific amino acid frequencies of somatic hypermutations at each complementarity-determining region position in heavy chain variable (VH) and κ light chain variable (Vκ) domain of the constructed library. The stacked column diagram shows the frequency and composition of mutations that occurred in each VH (**A**) and Vκ (**B**) sequence, respectively. The *x* axis shows the amino acid position of each variable domain, as defined by Kabat numbering.

We also identified the position-specific amino acid composition of non-germline residues at each CDR position in the VH or Vκ sequences (Figure 10). In HCDR1 and HCDR2, position H52c, which shows the highest frequency of SHM, had serine (5.9%) as the amino acid with the highest mutation ratio at that position, followed by threonine (4.8%) at H32, asparagine (4.9%) at H56, and asparagine (5.9%) at H31. (Figure 10A). In LCDR1, LCDR2, and LCDR3, position L93, which shows the highest SHM frequency, possessed threonine (8.7%) as the amino acid with the highest mutation ratio at that position, followed by asparagine (9.1%) at L31, aspartic acid (3.8%) at L92, and threonine (8.5%) at L53 (Figure 10B). Furthermore, we found that the most frequently substituted amino acids in HCDR1 and HCDR2, in descending order, were threonine (52.3%), serine (43.4%) and asparagine (42.1%), whereas those in LCDR1, LCDR2, and LCDR3 were threonine (62.4%), asparagine (49.6%), and serine (42.9%). In summary, threonine is one of the most frequently substituted amino acids in HCDRs and LCDRs.

### 2.6. Selection of Antigen-Specific Antibodies from the Constructed Library

In order to evaluate the quality of the constructed antibody library for usage as a high-quality antibody selection tool against proteins of interest, we performed biopanning to select scFvs against two therapeutically relevant target antigens, including one viral protein and one immune checkpoint protein: receptor binding domain (RBD) of severe acute respiratory syndrome coronavirus 2 (SARS-CoV-2) Omicron variant BA.2 and human and mouse CD155. After consecutive rounds of biopanning, positive scFv clones, specific to each target antigen, were finally selected using the phage ELISA. All the selected CD155-specific scFv clones were found to have strong reactivity to both human and mouse CD155, but not to bovine serum albumin (BSA), the negative control (Supplementary Figure S1). Through DNA sequencing, four SARS-CoV-2 BA.2 RBD-specific and four CD155-specific scFvs with different CDR sequences were identified. After the expression and purification of the scFvs using affinity column chromatography, the binding affinity to each target antigen was first measured using surface plasmon resonance (SPR) with Biacore T200. The results showed that the equilibrium dissociation constant (K_D_) values of the selected scFvs were determined in the nanomolar range (Table 1).

To compare the abundance of VH and Vκ subfamilies between the selected and original scFvs in the constructed library, both types of sequence data were analyzed. We found that the abundance of VH and Vκ subfamilies of the selected scFvs had similar patterns compared to those of the original scFvs in the constructed library (Supplementary Figure S2).

## 3. Discussion

The antibody library is a powerful tool for the discovery and development of target-specific antibodies for a wide range of applications. This includes not only research into the structure and function of proteins and pathological mechanisms of diseases but also into the development of diagnostics and therapeutic interventions [7,37]. Thus, the construction of a high-quality library with a larger and more diverse antibody repertoire is essential for increasing the chances of finding high-affinity antibodies with various paratopes that bind to the diverse epitopes of target antigens [38,39,40]. In this study, following in vitro immunization of hPBMCs, we constructed a large human combinatorial scFv library and comprehensively analyzed the characteristics of the newly constructed library using NGS. Finally, isolation and biochemical characterization of phage display-derived human scFvs specific to two therapeutically relevant target antigens, including the RBD of SARS-CoV-2 Omicron variant BA.2 and immune checkpoint protein CD155, were conducted for the quality assessment of the library.

Traditionally, mAbs with high affinity and selectivity have been generated from an antibody library, produced by the conventional immunization of animals with diverse antigens [7,41,42]. However, the method is labor-intensive and time-consuming and requires the additional establishment of a recombinant antibody library. Further, the mAbs derived from animals also have a higher immunogenicity risk, a major hurdle in the development of therapeutic antibodies [6]. To overcome this hurdle, a human naïve or synthetic antibody library has been constructed to isolate target-specific human antibodies [43,44,45]. Although vaccination in humanized mouse models is also used as another reliable method for the generation of human mAbs, based on our previous work and similar reports by others, phage display-based antibody selection from an established antibody library can considered a faster and more efficient approach to isolating human recombinant mAbs than the antibody selection from the immune library [8,46,47]. Therefore, we believe that the antibody phage display library generated in this study may be widely used as a novel rapid screening platform to select target-specific phage display-derived mAbs for use in multiple applications.

The naïve human antibody libraries have been constructed using human B cells of non-immunized or healthy donors [48]. The antibodies isolated from naïve human antibody libraries may often exhibit lower affinities because the antibody repertoire does not undergo in vivo affinity maturation to produce higher-affinity antibodies [48,49]. In the present study, we assert that the library we have developed is a high-quality human antibody library with large and diverse repertoires. Several lines of evidence support our notion. Firstly, antibody genes of each variable domain (VH or Vκ) were isolated from in vitro immunization of hPBMCs, particularly after stimulation by a combination of R848 and rhIL-2, and we found that AID, a marker protein used to initiate SHM, is highly upregulated. Thus, this suggests that the antibody gene repertoire in the library may be more diverse compared with the existing human naïve antibody library. Second, we constructed a large-sized combinatorial scFv library with 1.5 × 10^11^ antibody clones. Third, the library is comprised of approximately 94% and 91% of the unique antibody sequences in the VH and Vκ genes, respectively. Fourth, through two-dimensional heat maps illustrating VJ gene combinations in both VH and Vκ, we also confirmed that all the antibody genes in the library are almost evenly distributed. Fifth, comprehensive sequence analysis of the HCDRs and LCDRs showed that the library has more diverse amino acid composition in the CDRs than germline gene segments. Sixth, we found that the individual diversity at each position of the HCDRs and LCDRs in constructed library are approximately 4.3 times or 6.4 times higher than that in natural antibody sequences on average, respectively. Seventh, we observed that frequent SHMs occur in almost all amino acid positions of the library HCDRs and LCDRs and that some of them have a frequency of SHM over 25%. Further, the overall frequency of SHMs in HCDRs was relatively higher than that in LCDRs. Lastly, using phage display-based biopanning, we demonstrated that our library was able to yield various scFvs with K_D_ values in the nanomolar range that can be converted and generated to IgG antibodies having higher affinity [50]. Additionally, we could isolate the scFv antibodies that have cross-species reactivity in the nanomolar range of affinity from the constructed library. To the best of our knowledge, this is the first study to construct and validate a large human combinatorial antibody library with diverse antibody repertoires by employing an in vitro immunization setting to obtain unique antibody genes from stimulated hPBMCs.

The biophysiochemical properties of the antibody are critical for the effective development of a therapeutic antibody. Here, the developed antibody library has unique features. Through NGS analysis, we found that all the CDRs of the library lack a cysteine residue, which can undergo oxidation and form aberrant disulfide bonds that can affect antibody structure, stability, and biological function. In the constructed library, the most frequently occurring amino acids in HCDR3 were revealed to be tyrosine (13.7%), glycine (11.6%), and serine (7.0%), in that order. Birtalan et al. reported that tyrosine residues, combined with flexible glycine and serine residues in HCDR3, often contribute favorably to antigen recognition in high-affinity antibodies [51]. These findings suggest that the constructed antibody library may be used for the isolation of high-affinity antibodies. Unlike the VH gene usage dominated by VH3 and/or VH1 subfamilies in most human naïve antibody libraries [52,53], the most frequently found VH gene subfamilies in the library, in descending order, are VH4 and VH2. Furthermore, through phage display biopanning with two therapeutically relevant target antigens, we also found that most selected scFvs are mostly classified into one of VH4 and VH2 subfamilies, similar to what occurs with original scFvs in the library. Intriguingly, Shehata et al. reported that mAbs in the VH4 and VH2 subfamilies showed relatively higher thermostability than those in other subfamilies. In addition, the mAbs belonging to the VH4 subfamily showed low hydrophobicity, which was similar to mAbs belonging to the VH3 subfamily [54]. Therefore, these findings lead us to speculate that the newly constructed library may have the potential to discover high-quality antibodies.

In conclusion, this study demonstrated that, through the construction of a phage display antibody library using in vitro immunization protocol of hPBMC and consecutive NGS-based characterization of the library, a novel human antibody library with a large and diverse antibody repertoire can be generated. Furthermore, phage display biopanning also revealed that phage display-derived scFvs, isolated from the newly constructed antibody library, have a broad range of affinities to target antigens that are closely associated with viral diseases and cancer. In summary, these findings suggest that the library cannot only be used for the rapid development of target-specific phage display-derived mAbs, but can also provide fundamental insights into future strategies for antibody library construction with the use of functional paratope diversification. In the near future, we plan to perform in vivo efficacy and toxicological evaluations to validate the therapeutic potential of the selected phage display-derived mAbs.

## 4. Materials and Methods

### 4.1. Cell Culture

hPBMCs, purchased from different commercial sources (Lonza, Basel, Switzerland; StemCell Technologies, Vancouver, BC, Canada; iXCells Biotechnologies, San Diego, CA, USA; Zenbio, Inc., Durham, NC, USA), were maintained in a Roswell Park Memorial Institute 1640 (Gibco, Grand Island, NY, USA) medium, supplemented with 10% (*v*/*v*) fetal bovine serum (FBS, Gibco) and 1% (*v*/*v*) penicillin/streptomycin (Gibco) at 37 °C, with 5% CO_2_ and 95% humidity. For the immortalization of hPBMCs, the cells were infected with 250 μL of EBV (ATCC, Manassas, VA, USA) per 1 × 10^8^ hPBMCs. Throughout this study, the stimulation of hPBMCs was performed using a combination of 1 μg/mL R848 and 10 ng/mL rhIL-2 (Mabtech, Stockholm, Sweden). Cell counting of hPBMCs was performed using the ADAM-CellT instrument (NanoEntek inc., Seoul, Republic of Korea).

### 4.2. ELISA

For the measurement of Igs produced from the stimulated hPBMCs, the cell culture supernatant was obtained at every two days after the co-treatment of the cells with R848 and rhIL-2. Then, the concentration of each IgM and IgG was individually measured using a human IgM ELISA kit and human IgG ELISA kit (Abcam, Cambridge, UK) according to the manufacturer’s recommendations. Briefly, 50 μL of culture supernatant was added to the 96-well microtiter plate, pre-coated with an IgG or IgM as a capture antibody. Then, 50 μL of horseradish peroxidase (HRP)-conjugated IgG or IgM detector antibody was added to each well of the plate and incubated for 40 min at 25 °C. Following three washes with wash buffer, 3,3′,5,5′-tetramethylbenzidine (TMB) substrate solution (Thermo Fisher Scientific, Waltham, MA, USA) was added to each well and allowed to react with HRP for 5 min. The reaction was terminated by adding 100 μL of 1 M H_2_SO_4_. The absorbance of each sample was measured at 450 nm using a microplate reader (Bio-Tek Instruments, Winooski, VT, USA).

### 4.3. Immunoblot Analysis

To check the expression of AID in hPBMCs, the cells stimulated with or without a combination of R848 and rhIL-2 were harvested at 0, 2, and 4 days post-treatment. Following the performance of cell lysis with RIPA buffer (Thermo Fisher Scientific), protein extracts were quantified with the bicinchoninic acid protein assay kit (Thermo Fisher Scientific). Then, 20 μg of protein samples were subjected to sodium dodecyl sulfate-polyacrylamide gel electrophoresis and transferred to a nitrocellulose membrane (GE Healthcare, Chicago, IL, USA). After blocking in Tris-buffered saline with 0.1% (*v*/*v*) Tween 20 (TBS-T) containing 5% (*w*/*v*) BSA, the membrane was incubated with mouse anti-AID (Cell Signaling Technology) antibodies overnight at 4 °C. After several washes with TBS-T, the membranes were incubated with HRP-conjugated anti-mouse IgG antibody (Seracare Life Science, Milford, MA, USA). After multiple washes with TBS-T, the target proteins were visualized using SuperSignal West Pico PLUS Chemiluminescent Substrate (Thermo Fisher Scientific). Immunoreactive bands were detected using an Amersham^TM^ ImageQuant^TM^ 800 instrument (Cytiva, Marlborough, MA, USA).

### 4.4. Construction of Human Combinatorial Antibody Library

hPBMCs (2 × 10^8^), treated with R848 and rhIL-2 for 10 days, were harvested and lysed with 1 mL of Trizol reagent (Invitrogen, Waltham, MA, USA), followed by the addition of 0.2 mL of chloroform and incubation on ice for 2 min, for total RNA extraction. Following centrifugation at 12,000× *g* for 15 min, the aqueous phase was obtained. Total RNA was precipitated by the addition of 0.5 mL of isopropyl alcohol to this. After incubation on ice for 10 min and centrifugation at 12,000× *g* for 15 min, the resultant pellet was washed twice with 70% ethanol. Finally, the concentration of total RNA dissolved in DNase- and RNase-free water (Invitrogen) was measured using NanoDrop™ 2000/2000c Spectrophotometer (Thermo Fisher Scientific).

First-strand cDNA was synthesized from 5 µg of the total RNA template via reverse transcription using SuperScript™ III First-Strand Synthesis System (Invitrogen) and gene-specific primers: a primer for IgM VH 5′-GCAGGAGACGAGGGGGA-3′; a primer for IgG VH 5′-AGGGYGCCAGGGGGAA-3′; and a primer for Igκ 5′-AACAGAGGCAGTTCCAGA-3′. To amplify each VH or Vκ gene, PCR was performed with Q5 polymerase (New England Biolabs, Ipswich, MA, USA), 1 μL of cDNA template, and primer sets designed to obtain VH or Vκ germline genes. Then, each VH and Vκ PCR product was subjected to the overlap extension PCR to generate scFv sequences with Q5 polymerase (New England Biolabs) and primer sets containing a long linker sequence (5′-GGSSRSSSSGGGGSGGGG-3′) and SfiⅠ sites (5′-GGCCCAGGCGGCC-3′ and 5′-GGCCAGGCCGGCC-3′) (Supplementary Table S7). The thermal cycle conditions were as follows: initial denaturation at 98 °C for 3 min, 25 cycles at 98 °C for 10 s, 65 °C for 30 s, 72 °C for 40 s, and a final extension at 72 °C for 7 min. The final scFv PCR products were visualized on an agarose gel and purified using QIAquick Gel Extraction Kit (QIAGEN, Hilden, Germany).

The scFv PCR products were digested with SfiⅠ (New England Biolabs), cloned into the pComb3XSS phagemid vector, and transformed into *E. coli* K12 ER2738-competent cells via electroporation using a MicroPulser Electroporator (Bio-Rad Laboratories, Hercules, CA, USA). An aliquot of 100 μL was serially diluted and plated on LB-carbenicillin agar plates to measure the transformation titer. The transformed ER2738 cells were cultured in 1.6 L of super broth (SB) media (Super Broth; 3% *w*/*v* tryptone, 2% *w*/*v* yeast extract, and 1% *w*/*v* MOPS, pH 7.0) which had been supplemented with 50 μg/mL carbenicillin and 2% (*w*/*v*) glucose at 37 °C overnight. Following centrifugation at 5000× *g* for 20 min, the cell pellet was resuspended in 180 mL SB media with 20% glycerol and divided into 1.8 mL aliquots. The aliquots were stored at −80 °C.

### 4.5. NGS of the Antibody Library

Genes encoding VH and Vκ were amplified by PCR using the KAPA HiFi HotStart DNA polymerase (Roche, Basel, Switzerland), 84 ng of pComb3XSS phagemid vector encoding scFv genes as a template, and the following primers: forward and reverse primers for Vκ 5′-TGGCTGGTTTCGCTACC-3′ and 5′-GGAAGATCTAGAGGAACCACC-3′; forward and reverse primers for VH 5′-GGTGGTTCCTCTAGATCTTCC-3′ and 5′-TGATGGTGATGGTGCTGG-3′. The thermal cycle conditions were as follows: initial denaturation at 95 °C for 3 min, 25 cycles at 98 °C for 2 s, 60 °C for 15 s, 72 °C for 15 s, with a final extension at 72 °C for 1 min.

The PCR products were purified using CeleMag^TM^ Clean-up Bead (Celemics, Inc., Seoul, Republic of Korea) and concentrations were measured on a Tapestation 4200 instrument with D1000 ScreenTape (Agilent Technologies, Santa Clara, CA, USA). NGS libraries were prepared from 50 ng of the purified PCR products using a xGen^TM^ NGS Library Preparation kit (Integrated DNA Technologies, Inc., Coralville, IA, USA) according to the manufacturer’s recommendations. Briefly, the 3′ ends of PCR products were fragmented, blunted, and 5′ phosphorylated and were adenylated to facilitate their ligation to the sequencing adapters containing a single thymine overhang base pair. Then, adapter ligation was performed using a Ligation Master Mix. To amplify the adapter-ligated product, PCR was performed using a PCR Master Mix and the following primers containing the adapter sequences: forward primer 5’-GATCGGAAGAGCACACGTCTG

AACTCCAGTCACXXXXXXXXATCTCGTATGCCGTCTTCTGCTTG-3´ and reverse primer 5´-AATGATACGGCGACCACCGAGATCTACACYYYYYYYYACACTCTTTCCCTACA

CGACGCTCTTCCGATCT-3´.

Then, a quality control procedure was performed on the prepared NGS library using the microfluidic platform-based electrophoresis Tapestation 4200 instrument with the D1000 ScreenTape to confirm whether the length of the NGS library fragments was longer than 500 base pairs and whether the amount was over 5 ng/μL. Finally, equimolar concentrations of VH and Vκ NGS libraries were pooled and sequenced with a MiSeq instrument using a v3 300PE kit (Illumina Inc., San Diego, CA, USA).

### 4.6. Sequence Analysis of the Antibody Library

The forward and reverse reads of VH or Vκ, exhibiting varying overlap lengths from NGS raw data, were merged using Paired-End reAd mergeR (PEAR) software 0.9.8 (The Exelixis Lab, Heidelberg, Germany) with default parameters [55]. The VH or Vκ reads were aligned to germline sequences archived in the IMGT human antibody database using IgBLAST v1.8.0. The IgBLAST annotation was used for the identification of the closest-matching V and J germline gene segments for each VH or Vκ sequence. Then, incomplete sequences and problematic sequences (internal stop codon, frameshifts) were removed through multiple data processing steps and filters. Only a sequence containing all CDRs was used for further analysis.

### 4.7. Selection of Human scFvs from a Phage-Displayed Antibody Library

Biopanning was performed to isolate SARS-CoV-2 BA.2 RBD- and CD155-specific scFv antibodies from the constructed human antibody library. For the selection of SARS-CoV-2 BA.2 RBD-specific scFvs, 4 μg of recombinant SARS-CoV-2 BA.2 RBD protein (Sino Biological, Beijing, China) was coated onto magnetic beads (Dynabeads M-270 Epoxy; Invitrogen) according to the manufacturer’s instructions. Then, four rounds of biopanning were performed. For the selection of the CD155-specific scFvs with cross-species reactivity to human and mouse CD155, six rounds of biopanning were consecutively performed. Magnetic beads coated with 4 μg recombinant human CD155 (rhCD155) protein (Sino Biological) were used for the first and second biopanning rounds and recombinant mouse CD155 (rmCD155) protein (Sino Biological) for the third to sixth biopanning rounds. In each round, specific phages that bind to the target antigen were captured, and unbound phages were washed. Phage recovery and amplification were performed with helper phages (VCSM13). Ninety-six clones were randomly selected from the output colonies, and phage ELISA was conducted to test their reactivity to target antigens.

### 4.8. Phage ELISA

Individual colonies, selected from the third or fourth round of biopanning, were inoculated into 1 mL of SB media supplemented with 50 μg/mL of carbenicillin in 96-deep-well plates (Axygen, Union City, CA, USA) and incubated at 37 °C for 5 h. Then, 10^11^ pfu/mL of helper phages (VCSM13) and 70 μg/mL of kanamycin were added to the plates and incubated overnight at 37 °C. The plates were centrifuged at 3000× *g* for 30 min, and the phage supernatant was used for ELISA. Ninety-six-well high-binding microplates (Corning, NY, USA) were coated with 0.1 μg recombinant SARS-CoV-2 BA.2 RBD and rhCD155 (Sino Biological) and incubated overnight at 4 °C. After blocking using 3% (*w*/*v*) BSA in phosphate-buffered saline (PBS), the plates were incubated with 100 μL of phage supernatant at 37 °C for 2 h. After washing thrice with PBS containing 0.05% (*v/v*) Tween 20 (PBST), HRP-conjugated anti-hemagglutinin antibody (1:3000; Bethyl Laboratories, Montgomery, TX, USA) was added and incubated at 37 °C for 1 h. Colorimetric detection was performed by using the TMB substrate solution (Thermo Fisher Scientific, Waltham, MA, USA). The reaction was terminated by the addition of 1 M H_2_SO_4_, and absorbance was measured at 450 nm using a microtiter plate reader (Bio-Tek Instruments).

### 4.9. Production and Purification of Selected Human scFvs

Multiple colonies from *E. coli* BL21 (DE3)-harboring pComb3XSS phagemid vector-encoding scFv genes were cultured in the 3 mL SB media, supplemented with 50 μg/mL of carbenicillin, at 37 °C overnight. This starter culture was inoculated into 400 mL fresh SB media supplemented with 50 μg/mL carbenicillin and incubated at 37 °C until the OD_600_ value was 0.5. Isopropyl-β-d-thiogalactopyranoside (Bio Basic Inc., Markham, ON, Canada) was added to a 0.5 mM final concentration and the induced cells were grown at 37 °C for 4 h. Following centrifugation at 3000× *g* for 15 min, a periplasmic extract was obtained using the osmotic shock method [56]. Briefly, the cell pellets were resuspended with 10 mL of ice-cold 1× TES buffer (50 mM Tris–HCl, 1 mM EDTA, 20% sucrose, pH 8.0), and 15 mL of 0.2× TES buffer was subsequently added. After incubation on ice for 30 min, the suspension was centrifuged at 6000× *g* for 20 min and the resulting supernatant was obtained.

The periplasmic extract was incubated with 0.2 mL of HisPur^TM^ Ni-NTA resin (Thermo Fisher Scientific) for 1 h at 4 °C with slow rotation. The mixture was loaded onto a 2 mL polypropylene column (Thermo Fisher Scientific), and the agarose beads were washed with 2 mL of wash buffer (20 mM Tris-HCl, 200 mM NaCl, 5 mM imidazole, pH 7.5). ScFv was eluted with two 0.5 mL fractions of elution buffer (20 mM Tris-HCl, 200 mM NaCl, 500 mM imidazole, pH 7.5). To remove imidazole from the obtained protein solution and exchange the buffer with a physiological buffer, dialysis was performed in PBS using Slide-A-Lyzer dialysis cassettes (3500 Da MWCO, Thermo Fisher Scientific). The resulting scFvs were used for further analysis using SPR.

### 4.10. Determination of Binding Kinetics of Selected Target-Specific scFvs

The real-time interaction of antibody and antigen was measured using SPR with a Biacore T200 instrument (Cytiva). The carboxyl groups on the CM5 sensor chip (Cytiva) were activated with a mixture of 1-ethyl-3-(3-dimethylaminopropyl) carbodiimide and N-hydroxysuccinimide. The recombinant SARS-CoV-2 BA.2 RBD (Sino Biological) or rhCD155 (Sino Biological) was immobilized on an activated CM5 sensor chip for a read-out of up to 500 response units (RU). Unreacted carboxyl groups were blocked with 1 M ethanolamine (pH 8.5). Then, increasing concentrations (16, 32, 64, 128, and 256 nM) of the selected scFvs in the HBS-N buffer (10 mM HEPES, 150 mM NaCl, pH 7.4) were injected at a flow rate of 10 µL/min at 25 °C. Each association phase lasted 2 min and the dissociation phase lasted 10 min. To regenerate the sensor chips after each cycle, 10 mM glycine-HCl (pH 3.0) was injected to remove bound antibodies from the sensor chip surface. All the equilibrium dissociation constants (K_D_) were determined with the use of Biacore T200 Control Software Version 3.2 (Cytiva).

### 4.11. Statistical Analysis

The statistical analyses were carried out using GraphPad Prism (GraphPad Software Inc., San Diego, CA, USA). A two-tailed Student’s *t* test was used to conduct the comparisons between two groups. *p* Values of <0.05 were considered statistically significant (* *p* < 0.05; ** *p* < 0.01; *** *p* < 0.001). Graphs are presented as the mean of independent experiments ± standard error of the means (SEM).

## Figures and Tables

**Table 1:** Biochemical characterization of the selected phage display-derived single-chain variable fragments specific to therapeutically relevant target antigens.

Antigen	Antibody (scFv)	K_a_ (1/Ms)	K_d_ (1/s)	K_D_ (M)
SARS-CoV-2 BA.2 RBD	K105.1	3.30 × 10^5^	1.05 × 10^−3^	3.18 × 10^−9^
	K105.2	1.83 × 10^4^	1.35 × 10^−3^	7.40 × 10^−8^
	K105.3	7.03 × 10^4^	1.54 × 10^−3^	2.19 × 10^−8^
	K105.4	2.78 × 10^4^	1.79 × 10^−4^	6.44 × 10^−9^
Human CD155	K106.1	2.59 × 10^5^	1.46 × 10^−2^	5.66 × 10^−8^
	K106.2	1.22 × 10^6^	7.27 × 10^−2^	5.95 × 10^−8^
	K106.3	1.86 × 10^6^	8.91 × 10^−2^	4.79 × 10^−8^
	K106.4	1.53 × 10^4^	2.32 × 10^−3^	1.52 × 10^−7^

K_a_, association constant; K_d_, dissociation constant; K_D_, equilibrium dissociation constant.

## Data Availability

The datasets used and/or analyzed during the current study are available from the corresponding author on reasonable request.

## Supplementary Materials

The following supporting information can be downloaded at: https://www.mdpi.com/article/10.3390/ijms24066011/s1.
