# Supplementary material for: Generation and Next-Generation Sequencing-Based Characterization of a Large Human Combinatorial Antibody Library
Source: Int J Mol Sci. 2023 Mar 22;24(6):6011. doi: 10.3390/ijms24066011 (PMC10057307; doi:10.3390/ijms24066011)
Supplement: Supplementary file 1 [file ijms-24-06011-s001.zip › ijms-2217620-supplementary.pdf]

Generation and Next Generation Sequencing-based Characterization of a Large Human Combinatorial Antibody Library

Hye Lim Choi [1], Ha Rim Yang [1], Ha Gyeong Shin [1], Kyusang Hwang [1], Ji Woong Kim [2], Ji Hyun Lee [1], Taehoon Ryu [3], Yushin Jung [3] and Sukmook Lee [1,4,5,*]

[1] Department of Biopharmaceutical Chemistry, Kookmin University, Seoul 02707, Republic of Korea
[2] Department of Chemistry, Kookmin University, Seoul 02707, Republic of Korea
[3] ATG Lifetech Inc., Seoul 08507, Republic of Korea
[4] Department of Applied Chemistry, Kookmin University, Seoul 02707, Republic of Korea
[5] Antibody Research Institute, Kookmin University, Seoul 02707, Republic of Korea
* Correspondence: lees2018@kookmin.ac.kr; Tel.: +82-2-910-6763

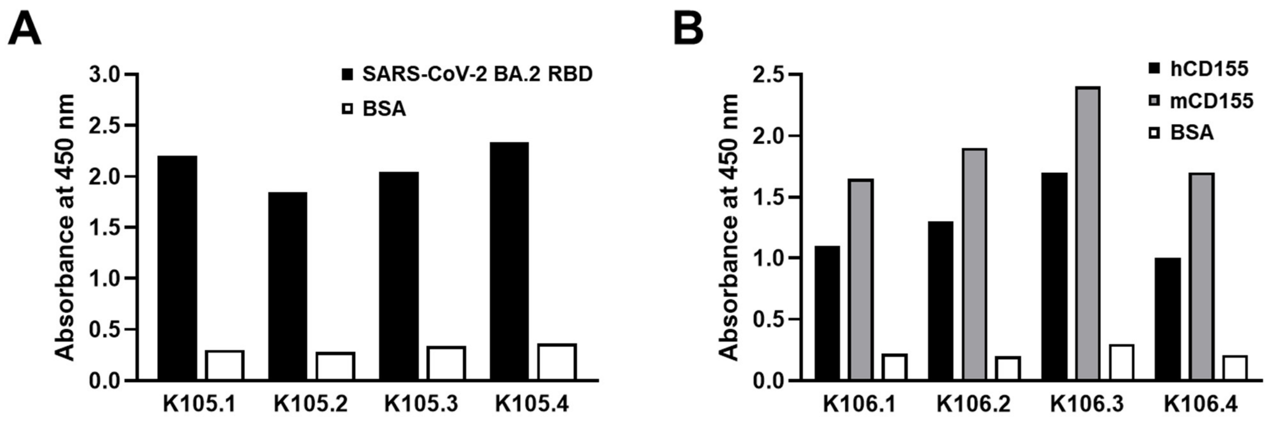

Supplementary Figure S1. Specific binding of the selected scFvs to target antigens. Phage ELISA was performed to verify the reactivity of the selected scFvs to SARS-CoV-2 BA.2 RBD (A) or the cross-species reactivity of the selected scFvs to human and mouse CD155 (hCD155 and mCD155) (B). Bovine serum albumin (BSA) was used as a negative control.

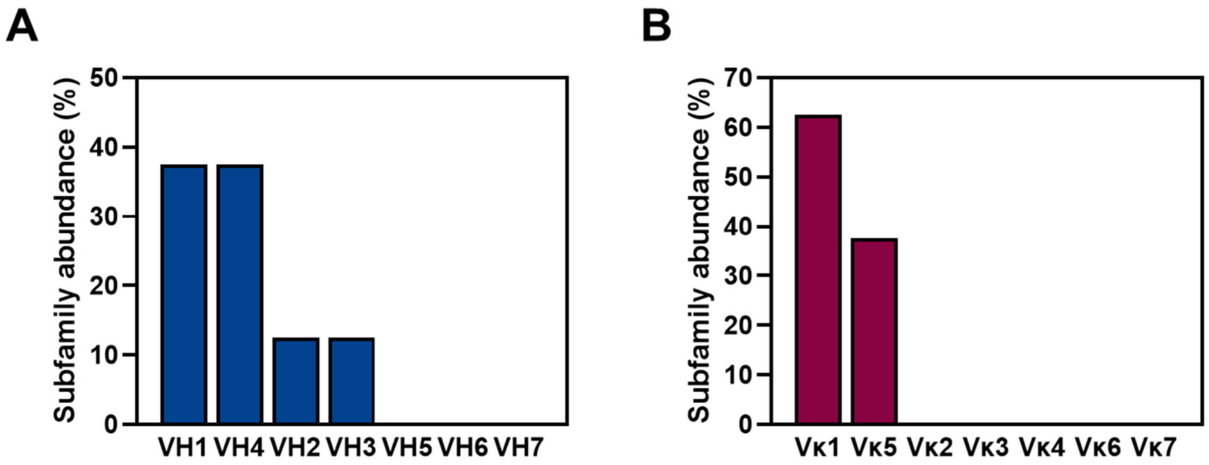

Supplementary Figure S2. Abundance of each variable domain subfamily observed in the scFvs selected from the constructed library. Following the germline classification of the antibody sequences selected from the library, each VH (A) and Vκ (B) subfamily was depicted as percentage bar graphs.

Supplementary Table S1. Primers used for the construction of human combinatorial antibody library.

Primer name	5′ to 3′ Sequence
Primers for VH germline genes	
**HSCVH1-FL**	GGTGGTTCCTCTAGATCTTCCTCCTCTGGTGGCGGTGGCTCGGGCGGTGGTGGGCAGGTGCAGCTGGTGCAGTCTGG
**HSCVH2-FL**	GGTGGTTCCTCTAGATCTTCCTCCTCTGGTGGCGGTGGCTCGGGCGGTGGTGGGCAGATCACCTTGAAGGAGTCTGG
**HSCVH35-FL**	GGTGGTTCCTCTAGATCTTCCTCCTCTGGTGGCGGTGGCTCGGGCGGTGGTGGGGAGGTGCAGCTGGTGSAGTCTGG
**HSCVH3a-FL**	GGTGGTTCCTCTAGATCTTCCTCCTCTGGTGGCGGTGGCTCGGGCGGTGGTGGGGAGGTGCAGCTGKTGGAGTCTG
**HSCVH4-FL**	GGTGGTTCCTCTAGATCTTCCTCCTCTGGTGGCGGTGGCTCGGGCGGTGGTGGGCAGGTGCAGCTGCAGGAGTCGGG
**HSCVH4a-FL**	GGTGGTTCCTCTAGATCTTCCTCCTCTGGTGGCGGTGGCTCGGGCGGTGGTGGGCAGGTGCAGCTACAGCAGTGGGG
**VH1 Fw**	GGTGGTTCCTCTAGATCTTCCTCCTCTGGTGGCGGTGGCTCGGGCGGTGGTGGGCAGGTCCAGCTKGTRCAGTCTGG
**VH1 Fw_2**	GGTGGTTCCTCTAGATCTTCCTCCTCTGGTGGCGGTGGCTCGGGCGGTGGTGGGCAGGTGCAGCTGGTGCAATCTGG
**VH157 Fw**	GGTGGTTCCTCTAGATCTTCCTCCTCTGGTGGCGGTGGCTCGGGCGGTGGTGGGCAGGTGCAGCTGGTGGARTCTGG
**VH2 Fw**	GGTGGTTCCTCTAGATCTTCCTCCTCTGGTGGCGGTGGCTCGGGCGGTGGTGGGCAGGTCACCTTGAAGGAGTCTG
**VH4 Fw**	GGTGGTTCCTCTAGATCTTCCTCCTCTGGTGGCGGTGGCTCGGGCGGTGGTGGGCAGGTGCAGCTGCAGGAGTCCG
**VH6 Fw**	GGTGGTTCCTCTAGATCTTCCTCCTCTGGTGGCGGTGGCTCGGGCGGTGGTGGGCAGGTACAGCTGCAGCAGTCA
**HSCG1234-B**	CCTGGCCGGCCTGGCCACTAGTGACCGATGGGCCCTTGGTGGARG
**HSCM-B**	CCTGGCCGGCCTGGCCACTAGTAAGGGTTGGGGCGGATGCACTCCC
Primers for Vκ germline genes	
**HSCK1-F**	GGGCCCAGGCGGCCGAGCTCCAGATGACCCAGTCTCC
**HSCK24-F**	GGGCCCAGGCGGCCGAGCTCGTGATGACYCAGTCTCC
**HSCK3-F**	GGGCCCAGGCGGCCGAGCTCGTGWTGACRCAGTCTCC
**HSCK5-F**	GGGCCCAGGCGGCCGAGCTCACACTCACGCAGTCTCC
**VK1 Fw**	GGGCCCAGGCGGCCGACATCCRGDTGACCCAGTCTCC
**VK3 Fw**	GGGCCCAGGCGGCCGAAATTGTRWTGACRCAGTCTCC
**VK5 Fw**	GGGCCCAGGCGGCCGAAACGACACTCACGCAGTCTC
**VK246 Fw**	GGGCCCAGGCGGCCGATATTGTGMTGACBCAGWCTCC
**Vk-2 Fw**	GGGCCCAGGCGGCCGATGTTGTGATGACYCAG
**Vk-4 Fw**	GGGCCCAGGCGGCCGACATCGTGATGACCCAGT
**Vk-1 Fw_2**	GGGCCCAGGCGGCCGCCATCCGGTTGACCCAGT
**HSCJK14o-B**	GGAAGATCTAGAGGAACCACCTTTGATSTCCACCTTGGTCCC
**HSCJK2o-B**	GGAAGATCTAGAGGAACCACCTTTGATCTCCAGCTTGGTCCC
**HSCJK3o-B**	GGAAGATCTAGAGGAACCACCTTTGATATCCACTTTGGTCCC
**HSCJK5o-B**	GGAAGATCTAGAGGAACCACCTTTAATCTCCAGTCGTGTCCC

Supplementary Table S2. The percentage of pairing of germline V and J gene segments in each VH and $V\kappa$ of the constructed library.

VH	IGHJ 1-01	IGHJ 2-01	IGHJ 3-01	IGHJ 3-02	IGHJ 4-01	IGHJ 4-02	IGHJ 4-03	IGHJ 5-01	IGHJ 5-02	IGHJ 6-01	IGHJ 6-02	IGHJ 6-03	IGHJ 6-04
**IGHV1-18**	0.012	0.009	0.019	0.085	0.003	0.350	0.001	0.003	0.078	0.001	0.105	0.043	0.002
**IGHV1-2**	0.017	0.012	0.028	0.099	0.004	0.573	0.002	0.006	0.170	0.003	0.151	0.061	0.001
**IGHV1-24**	0.003	0.004	0.004	0.027	0.000	0.049	0.000	0.000	0.019	0.000	0.030	0.007	0.000
**IGHV1-3**	0.005	0.008	0.017	0.037	0.003	0.194	0.001	0.003	0.082	0.000	0.054	0.037	0.001
**IGHV1-38-4**	0.000	0.000	0.000	0.000	0.000	0.000	0.000	0.000	0.000	0.000	0.000	0.000	0.000
**IGHV1-45**	0.000	0.000	0.000	0.008	0.000	0.001	0.000	0.000	0.000	0.000	0.002	0.000	0.000
**IGHV1-46**	0.013	0.010	0.011	0.094	0.003	0.387	0.001	0.004	0.088	0.001	0.080	0.030	0.000
**IGHV1-58**	0.000	0.000	0.000	0.001	0.000	0.008	0.000	0.000	0.002	0.000	0.003	0.001	0.000
**IGHV1-69**	0.044	0.065	0.064	0.239	0.007	0.745	0.002	0.014	0.214	0.003	0.388	0.148	0.002
**IGHV1-69-2**	0.000	0.000	0.000	0.001	0.000	0.009	0.000	0.000	0.001	0.000	0.002	0.000	0.000
**IGHV1-8**	0.055	0.014	0.006	0.040	0.001	0.176	0.009	0.001	0.054	0.000	0.048	0.030	0.000
**IGHV1/OR15-1**	0.000	0.000	0.000	0.000	0.000	0.000	0.000	0.000	0.000	0.000	0.000	0.000	0.000
**IGHV1/OR15-2**	0.000	0.000	0.000	0.000	0.000	0.000	0.000	0.000	0.000	0.000	0.000	0.000	0.000
**IGHV1/OR15-3**	0.000	0.000	0.000	0.000	0.000	0.000	0.000	0.000	0.000	0.000	0.000	0.000	0.000
**IGHV2-26**	0.028	1.005	0.100	0.412	0.009	1.968	0.003	0.018	1.493	0.007	1.150	0.218	0.004
**IGHV2-5**	0.293	0.486	0.562	1.893	0.097	13.331	0.236	0.147	2.455	0.003	1.274	0.448	0.009
**IGHV2-70**	0.008	0.025	0.094	0.293	0.011	0.984	0.002	0.033	0.340	0.001	0.269	0.106	0.002
**IGHV2-70D**	0.004	0.041	0.097	0.426	0.008	1.424	0.002	0.046	0.218	0.001	0.389	0.060	0.005
**IGHV3-11**	0.005	0.013	0.015	0.041	0.002	0.216	0.000	0.004	0.028	0.000	0.044	0.022	0.001
**IGHV3-13**	0.001	0.019	0.002	0.011	0.000	0.036	0.000	0.000	0.006	0.000	0.012	0.004	0.000
**IGHV3-15**	0.008	0.007	0.006	0.028	0.001	0.196	0.002	0.002	0.025	0.000	0.047	0.011	0.000
**IGHV3-20**	0.000	0.002	0.001	0.003	0.000	0.018	0.000	0.000	0.004	0.000	0.004	0.004	0.000
**IGHV3-21**	0.003	0.006	0.008	0.031	0.002	0.160	0.000	0.002	0.024	0.000	0.041	0.017	0.000
**IGHV3-22**	0.000	0.000	0.000	0.000	0.000	0.000	0.000	0.000	0.000	0.000	0.000	0.000	0.000
**IGHV3-23**	0.021	0.035	0.030	0.112	0.012	0.737	0.001	0.012	0.091	0.000	0.133	0.050	0.001
**IGHV3-30**	0.026	0.028	0.026	0.095	0.007	0.648	0.001	0.007	0.086	0.000	0.134	0.054	0.001
**IGHV3-30-3**	0.010	0.007	0.008	0.049	0.002	0.193	0.001	0.001	0.035	0.000	0.066	0.016	0.000
**IGHV3-33**	0.007	0.032	0.007	0.074	0.004	0.269	0.001	0.003	0.039	0.001	0.075	0.008	0.000
**IGHV3-35**	0.000	0.000	0.000	0.000	0.000	0.000	0.000	0.000	0.000	0.000	0.000	0.000	0.000
**IGHV3-43**	0.001	0.002	0.003	0.003	0.001	0.049	0.000	0.001	0.006	0.000	0.009	0.008	0.000
**IGHV3-43D**	0.001	0.001	0.002	0.003	0.001	0.025	0.000	0.001	0.004	0.000	0.015	0.004	0.000
**IGHV3-47**	0.000	0.000	0.000	0.000	0.000	0.000	0.000	0.000	0.000	0.000	0.000	0.000	0.000
**IGHV3-48**	0.007	0.014	0.015	0.055	0.004	0.280	0.000	0.005	0.041	0.000	0.052	0.025	0.000
**IGHV3-49**	0.007	0.005	0.003	0.018	0.001	0.126	0.000	0.003	0.020	0.000	0.024	0.018	0.000
**IGHV3-52**	0.000	0.000	0.000	0.000	0.000	0.000	0.000	0.000	0.000	0.000	0.000	0.000	0.000
**IGHV3-53**	0.004	0.008	0.008	0.030	0.001	0.110	0.000	0.001	0.016	0.000	0.032	0.010	0.000
**IGHV3-64**	0.001	0.002	0.002	0.004	0.000	0.031	0.000	0.000	0.004	0.000	0.005	0.002	0.000
**IGHV3-64D**	0.000	0.004	0.001	0.004	0.000	0.028	0.000	0.001	0.002	0.000	0.012	0.000	0.000
**IGHV3-66**	0.004	0.004	0.006	0.015	0.001	0.073	0.000	0.001	0.008	0.000	0.012	0.010	0.000
**IGHV3-69-1**	0.000	0.000	0.000	0.000	0.000	0.003	0.000	0.000	0.001	0.000	0.001	0.000	0.000
**IGHV3-7**	0.012	0.009	0.007	0.033	0.002	0.231	0.000	0.003	0.034	0.000	0.044	0.021	0.000
**IGHV3-71**	0.000	0.000	0.000	0.000	0.000	0.001	0.000	0.000	0.000	0.000	0.000	0.000	0.000
**IGHV3-72**	0.008	0.005	0.006	0.026	0.002	0.073	0.000	0.000	0.006	0.000	0.013	0.005	0.000
**IGHV3-73**	0.001	0.003	0.003	0.015	0.002	0.075	0.000	0.000	0.014	0.000	0.008	0.007	0.000
**IGHV3-74**	0.004	0.011	0.008	0.033	0.001	0.177	0.000	0.002	0.030	0.000	0.048	0.010	0.000
**IGHV3-9**	0.019	0.032	0.030	0.112	0.003	0.439	0.004	0.006	0.050	0.000	0.095	0.047	0.000
**IGHV3-NL1**	0.001	0.001	0.001	0.003	0.000	0.016	0.000	0.000	0.002	0.000	0.003	0.001	0.000
**IGHV3/OR16-10**	0.000	0.000	0.000	0.000	0.000	0.000	0.000	0.000	0.000	0.000	0.000	0.000	0.000
**IGHV3/OR16-8**	0.000	0.000	0.000	0.000	0.000	0.000	0.000	0.000	0.000	0.000	0.000	0.000	0.000
**IGHV4-28**	0.000	0.002	0.000	0.006	0.000	0.017	0.000	0.001	0.008	0.000	0.005	0.001	0.000
**IGHV4-30-2**	0.003	0.014	0.010	0.025	0.002	0.118	0.000	0.002	0.040	0.000	0.020	0.005	0.000

**IGHV4-30-4**	0.008	0.034	0.017	0.079	0.008	0.386	0.001	0.011	0.118	0.000	0.076	0.037	0.001
**IGHV4-31**	0.011	0.067	0.048	0.164	0.027	0.673	0.002	0.013	0.174	0.001	0.283	0.019	0.001
**IGHV4-34**	0.188	0.715	0.434	1.062	0.073	10.667	0.013	0.053	2.424	0.024	2.629	0.480	0.011
**IGHV4-38-2**	0.046	0.124	0.053	0.229	0.018	1.116	0.003	0.014	0.313	0.001	0.181	0.079	0.002
**IGHV4-39**	0.092	0.204	0.097	0.574	0.038	3.059	0.009	0.048	0.983	0.002	0.679	0.212	0.003
**IGHV4-4**	0.024	0.058	0.046	0.189	0.015	1.089	0.002	0.024	0.285	0.001	0.294	0.035	0.001
**IGHV4-55**	0.000	0.000	0.000	0.000	0.000	0.001	0.000	0.000	0.000	0.000	0.000	0.000	0.000
**IGHV4-59**	0.035	0.281	0.079	0.390	0.018	1.447	0.003	0.068	0.507	0.001	0.537	0.113	0.002
**IGHV4-61**	0.017	0.091	0.030	0.159	0.008	0.835	0.001	0.033	0.204	0.001	0.195	0.070	0.004
**IGHV4/OR15-8**	0.000	0.001	0.000	0.001	0.000	0.015	0.000	0.001	0.001	0.000	0.002	0.000	0.000
**IGHV5-10-1**	0.001	0.006	0.005	0.029	0.001	0.053	0.000	0.000	0.017	0.000	0.036	0.001	0.002
**IGHV5-51**	0.019	0.039	0.046	0.266	0.005	0.615	0.002	0.009	0.140	0.000	0.151	0.051	0.001
**IGHV6-1**	0.478	0.456	0.216	1.917	0.039	8.102	0.011	0.061	1.228	0.003	2.979	0.124	0.005
**IGHV7-4-1**	0.007	0.013	0.020	0.036	0.001	0.264	0.002	0.003	0.067	0.000	0.044	0.035	0.000
**IGHV7-81**	0.000	0.000	0.000	0.000	0.000	0.000	0.000	0.000	0.000	0.000	0.000	0.000	0.000

**Vκ**	**IGKJ1-01**	**IGKJ2-01**	**IGKJ2-02**	**IGKJ2-03**	**IGKJ2-04**	**IGKJ3-01**	**IGKJ4-01**	**IGKJ4-02**	**IGKJ5-01**
**IGKV1-12**	0.099	0.219	0.005	0.005	0.001	0.431	0.657	0.009	1.021
**IGKV1-13**	0.032	0.073	0.001	0.001	0.000	0.337	0.562	0.001	1.128
**IGKV1-16**	0.089	0.139	0.003	0.003	0.001	0.355	0.448	0.002	0.872
**IGKV1-17**	0.150	0.165	0.006	0.004	0.001	0.208	0.512	0.003	0.333
**IGKV1-27**	0.120	0.080	0.002	0.002	0.000	0.617	0.535	0.002	0.319
**IGKV1-33**	0.069	0.500	0.009	0.017	0.005	1.198	1.646	0.006	2.321
**IGKV1-37**	0.004	0.021	0.000	0.009	0.000	0.021	0.047	0.000	0.199
**IGKV1-39**	0.635	1.099	0.035	0.034	0.012	1.617	1.551	0.012	2.776
**IGKV1-5**	0.627	0.507	0.017	0.024	0.008	0.347	0.749	0.005	0.617
**IGKV1-6**	0.123	0.128	0.002	0.001	0.000	0.069	0.241	0.001	0.080
**IGKV1-8**	0.142	0.146	0.002	0.001	0.001	0.395	0.525	0.003	0.564
**IGKV1-9**	0.104	0.290	0.008	0.010	0.003	0.919	0.958	0.005	2.695
**IGKV1-NL1**	0.068	0.078	0.002	0.001	0.000	0.093	0.137	0.000	0.545
**IGKV1/OR-3**	0.000	0.000	0.000	0.000	0.000	0.000	0.000	0.000	0.000
**IGKV1/OR-4**	0.000	0.000	0.000	0.000	0.000	0.000	0.000	0.000	0.000
**IGKV1/OR10-1**	0.000	0.000	0.000	0.000	0.000	0.000	0.000	0.000	0.000
**IGKV1/OR2-1**	0.000	0.000	0.000	0.000	0.000	0.000	0.000	0.000	0.000
**IGKV1/OR2-108**	0.000	0.000	0.000	0.000	0.000	0.000	0.001	0.000	0.000
**IGKV1D-12**	0.010	0.041	0.002	0.000	0.000	0.089	0.137	0.001	0.246
**IGKV1D-13**	0.009	0.072	0.000	0.000	0.000	0.087	0.109	0.000	0.093
**IGKV1D-16**	0.018	0.027	0.001	0.001	0.000	0.046	0.087	0.000	0.205
**IGKV1D-17**	0.000	0.000	0.000	0.000	0.000	0.000	0.000	0.000	0.000
**IGKV1D-43**	0.003	0.005	0.000	0.000	0.000	0.115	0.026	0.000	0.093
**IGKV1D-8**	0.005	0.007	0.000	0.000	0.000	0.003	0.007	0.000	0.010
**IGKV2-18**	0.000	0.000	0.000	0.000	0.000	0.001	0.001	0.000	0.001
**IGKV2-24**	0.069	0.196	0.003	0.002	0.000	0.152	0.181	0.000	0.489
**IGKV2-28**	0.244	0.547	0.054	0.007	0.005	1.364	1.022	0.005	2.025
**IGKV2-29**	0.020	0.051	0.002	0.001	0.000	0.078	0.126	0.000	0.189
**IGKV2-30**	0.340	0.622	0.016	0.008	0.002	1.339	0.849	0.002	2.042
**IGKV2-4**	0.000	0.000	0.000	0.000	0.000	0.000	0.000	0.000	0.000
**IGKV2-40**	0.011	0.059	0.001	0.007	0.000	0.034	0.051	0.000	0.156
**IGKV2D-18**	0.000	0.000	0.000	0.000	0.000	0.000	0.000	0.000	0.000
**IGKV2D-24**	0.001	0.001	0.000	0.000	0.000	0.001	0.001	0.000	0.002
**IGKV2D-26**	0.001	0.001	0.000	0.000	0.000	0.001	0.009	0.000	0.001
**IGKV2D-29**	0.148	0.302	0.004	0.003	0.001	0.724	0.810	0.002	0.831
**IGKV2D-30**	0.017	0.039	0.000	0.001	0.000	0.091	0.058	0.000	0.206
**IGKV3-11**	0.102	0.182	0.005	0.003	0.005	0.874	1.164	0.003	1.640

**IGKV3-15**	0.462	0.723	0.022	0.012	0.005	0.848	1.449	0.014	1.641
**IGKV3-20**	1.164	1.695	0.056	0.030	0.009	2.969	2.976	0.009	3.316
**IGKV3-7**	0.000	0.000	0.000	0.000	0.000	0.001	0.000	0.000	0.001
**IGKV3/OR2-268**	0.004	0.008	0.000	0.000	0.000	0.011	0.016	0.000	0.016
**IGKV3D-11**	0.001	0.003	0.000	0.000	0.000	0.007	0.006	0.000	0.022
**IGKV3D-15**	0.049	0.082	0.003	0.001	0.001	0.076	0.192	0.002	0.359
**IGKV3D-20**	0.065	0.108	0.004	0.001	0.001	0.274	0.405	0.002	0.496
**IGKV4-1**	1.555	2.195	0.058	0.026	0.023	4.017	5.313	0.027	6.065
**IGKV5-2**	0.701	1.520	0.013	0.175	0.003	1.413	1.012	0.004	0.120
**IGKV6-21**	0.008	0.015	0.000	0.000	0.000	0.027	0.033	0.000	0.209
**IGKV6D-21**	0.003	0.007	0.000	0.001	0.000	0.003	0.008	0.000	0.015
**IGKV6D-41**	0.000	0.000	0.000	0.000	0.000	0.000	0.001	0.000	0.000
**IGKV7-3**	0.000	0.001	0.000	0.000	0.000	0.000	0.002	0.000	0.000

The percentages of all the pairing of germline V and J gene segments having cut-off ≥ 0.001 are represented. In case of the pairing of the gene segments cut-off < 0.001 were expressed as 0.000.

Supplementary Table S3. The percentage of amino acid composition at each position in HCDRs of the constructed library.

Position	A	C	D	E	F	G	H	I	K	L	M	N	P	Q	R	S	T	V	W	Y
**H31**	1.58	0.10	4.97	0.32	0.06	16.58	0.27	0.68	0.29	0.06	0.05	9.96	0.40	0.03	1.83	36.73	25.66	0.20	0.00	0.23
**H32**	4.88	0.20	1.81	0.19	1.38	7.54	2.87	0.64	0.28	0.38	0.02	15.03	1.31	0.09	1.86	23.85	4.80	0.34	0.01	32.52
**H33**	7.13	0.24	2.78	1.19	1.20	27.60	0.82	0.62	0.67	0.29	0.03	3.30	0.99	0.21	5.66	16.94	3.12	0.63	3.21	23.34
**H34**	11.09	0.43	0.37	0.79	1.11	1.40	0.76	6.67	0.03	2.09	20.28	0.26	0.10	0.05	0.13	0.74	0.80	19.79	21.61	11.49
**H35**	12.28	2.13	0.49	0.02	0.70	24.31	7.54	0.41	0.05	0.15	0.05	4.87	0.18	0.22	3.29	22.90	5.22	0.70	4.73	9.77
**H35a**	0.53	0.39	0.07	0.03	0.05	4.13	0.02	0.08	0.00	0.83	0.43	0.31	0.02	0.00	0.21	2.90	0.52	48.67	40.79	0.02
**H35b**	3.58	0.22	0.85	0.00	0.04	43.06	1.05	0.41	0.03	0.04	0.00	23.34	0.04	0.00	0.31	23.23	2.72	0.61	0.00	0.47
**H50**	1.29	0.21	0.78	17.58	1.91	3.89	6.13	3.29	1.09	16.73	0.27	2.03	0.04	0.46	19.57	7.73	1.79	4.33	2.90	7.99
**H51**	0.28	0.01	0.01	0.00	1.06	0.12	0.01	75.92	0.05	1.37	1.68	0.13	0.02	0.00	0.06	0.65	14.23	4.38	0.00	0.03
**H52**	0.42	0.17	6.14	0.11	8.17	0.42	2.52	2.32	1.19	0.49	0.04	18.46	0.04	0.17	1.27	8.64	1.11	0.22	0.62	47.46
**H52a**	3.45	0.12	0.71	0.36	2.97	5.35	1.36	0.21	0.18	0.28	0.01	1.32	22.74	1.22	0.65	7.78	3.21	0.46	3.83	43.78
**H52b**	0.16	0.04	0.31	0.35	0.03	1.94	0.01	0.12	7.38	0.64	5.03	0.55	0.01	0.18	79.53	1.69	1.47	0.04	0.48	0.05
**H52c**	40.34	0.01	3.76	0.25	0.51	5.69	0.37	2.09	0.46	0.65	0.02	2.20	4.02	0.00	1.03	9.20	25.80	3.49	0.01	0.11
**H53**	0.49	0.12	5.37	0.39	0.53	2.83	19.22	1.60	0.32	0.43	0.29	4.28	0.93	0.86	0.73	22.96	0.91	0.32	24.31	13.11
**H54**	0.53	0.04	21.72	0.56	1.95	9.87	0.24	0.56	11.65	0.39	0.04	12.15	0.05	0.38	2.35	32.97	4.09	0.22	0.01	0.23
**H55**	0.79	0.04	27.66	0.91	0.07	47.65	0.14	0.10	0.08	0.05	0.02	1.29	0.05	0.01	0.66	5.43	0.64	0.35	13.60	0.45
**H56**	1.44	0.14	25.47	6.49	0.77	2.78	1.25	1.41	0.96	0.18	0.06	8.33	0.15	0.33	2.24	29.30	6.94	0.63	0.01	11.12
**H57**	3.35	0.00	0.23	1.47	0.29	0.14	0.44	3.01	28.54	0.12	0.15	10.00	1.57	0.65	1.19	2.30	44.76	0.49	0.00	1.29
**H58**	0.61	0.07	13.93	2.11	3.86	1.54	2.22	0.73	2.26	0.25	0.10	24.11	0.14	0.31	20.35	7.00	1.86	0.44	0.05	18.07
**H59**	0.01	0.19	0.30	0.01	1.25	0.05	1.12	0.08	0.05	0.15	0.00	0.46	0.05	0.14	0.03	1.06	0.04	0.07	0.00	94.92
**H60**	27.95	0.04	0.32	0.22	0.02	0.65	0.26	0.48	0.55	0.18	0.07	35.21	0.31	0.03	1.74	29.09	1.78	1.03	0.00	0.07
**H61**	2.40	0.00	8.31	1.12	0.07	0.67	0.13	0.33	0.11	2.00	0.07	0.09	57.18	7.00	0.31	1.45	9.66	9.07	0.02	0.01
**H62**	0.64	0.02	0.10	0.05	0.59	0.59	0.04	0.02	5.71	0.07	0.04	0.62	0.98	0.09	0.40	89.36	0.48	0.02	0.00	0.18
**H63**	0.13	0.01	0.00	0.05	8.89	0.05	0.11	0.14	0.00	66.80	0.65	0.00	0.17	0.10	0.12	0.04	0.01	22.67	0.01	0.03
**H64**	0.22	0.00	0.11	3.02	0.00	0.18	0.12	0.02	74.73	0.10	0.37	1.36	0.02	10.80	7.30	0.38	1.16	0.05	0.04	0.02
**H65**	0.17	0.07	0.87	0.38	0.02	21.71	0.06	0.12	0.02	0.01	0.01	2.94	0.13	0.00	1.26	65.33	6.82	0.04	0.01	0.01
**H95**	6.36	0.45	12.36	5.68	1.11	18.56	3.11	4.86	1.52	4.24	1.37	1.32	1.49	2.46	12.94	8.04	5.89	5.82	1.00	1.42
**H96**	4.61	0.42	4.08	3.69	2.05	12.97	2.34	3.57	2.38	7.80	0.86	2.60	10.05	3.04	13.84	8.12	5.87	4.98	1.84	4.89
**H97**	5.83	1.65	5.14	2.80	3.14	17.11	2.91	3.51	1.44	4.78	1.32	2.81	5.73	1.72	8.57	9.63	5.61	5.84	1.87	8.59
**H98**	7.29	1.34	6.15	2.40	2.80	16.77	1.44	3.39	0.69	3.68	1.17	3.27	3.55	1.53	5.99	11.66	6.81	6.37	3.25	10.45
**H99**	7.98	1.33	5.40	2.07	3.60	15.78	1.59	2.39	0.92	4.64	1.07	3.23	2.76	1.27	4.80	14.47	6.97	5.12	2.97	11.65
**H100**	7.41	0.95	6.13	1.81	5.16	16.17	1.38	2.86	1.29	4.04	1.11	2.80	2.69	1.07	4.24	14.05	5.77	5.56	5.29	10.22
**H100a**	6.82	1.48	6.16	1.61	6.92	15.29	2.18	2.15	0.90	5.44	1.42	3.19	4.21	1.02	3.81	12.80	5.25	4.38	3.52	11.44
**H100b**	5.81	1.93	4.50	1.58	9.81	15.07	1.47	2.30	0.91	3.96	1.42	3.80	3.88	1.03	3.92	10.81	4.76	4.30	6.70	12.05
**H100c**	5.34	1.74	4.68	1.41	12.01	11.96	2.10	2.25	1.00	5.85	1.38	3.46	4.02	0.70	3.72	8.62	4.42	4.53	5.13	15.67
**H100d**	5.97	2.70	4.18	2.03	12.45	11.04	2.25	1.59	1.08	4.11	1.60	3.14	4.20	0.61	3.54	7.84	3.88	3.79	4.42	19.59
**H100e**	3.79	1.63	3.44	0.86	17.66	10.28	2.23	1.31	0.98	5.26	3.27	3.18	3.69	1.17	4.26	6.08	2.26	2.55	6.91	19.18
**H100f**	4.51	1.77	3.67	0.93	18.58	9.98	1.55	1.32	1.19	4.14	5.08	3.82	3.95	0.57	4.07	5.18	1.83	1.96	3.57	22.33
**H100g**	4.55	0.40	2.92	0.82	21.78	12.18	3.61	1.54	1.38	3.91	5.07	2.44	3.19	0.68	3.16	3.02	2.51	2.26	3.97	20.60
**H100h**	5.27	0.38	2.27	1.59	25.47	11.06	1.69	1.08	1.12	3.49	7.26	2.64	2.91	0.81	1.98	2.97	2.07	1.40	3.65	20.89
**H100i**	4.78	0.53	2.64	0.43	24.58	12.93	1.68	1.33	0.27	3.62	11.45	4.29	1.67	0.24	0.65	1.97	0.82	1.81	4.86	19.43
**H100j**	5.30	0.25	0.30	0.06	32.66	9.95	0.73	0.60	0.06	4.46	21.95	0.26	1.26	0.06	0.38	0.76	0.64	1.23	6.51	12.61
**H100k**	0.05	0.30	0.04	0.03	61.53	0.07	0.03	1.72	0.04	4.56	28.04	0.04	0.22	0.05	0.07	0.76	0.27	1.93	0.07	0.21
**H101**	1.34	0.03	85.50	1.97	0.30	3.32	1.00	0.14	0.16	0.90	0.06	0.97	0.45	0.99	0.41	0.69	0.38	0.61	0.17	0.62
**H102**	0.47	0.58	1.22	0.18	3.61	0.23	2.50	10.22	0.13	4.19	0.37	1.43	11.23	0.21	0.28	5.87	0.66	17.55	0.07	39.00

The percentages of the amino acid composition at each position in HCDRs having cut-off ≥ 0.01 are represented. In case of the amino acid compositions cut-off < 0.01 were expressed as 0.00.

Supplementary Table S4. The average percentage of amino acid composition in HCDR3 of the constructed library.

Amino acid composition (%)																			
**A**	**C**	**D**	**E**	**F**	**G**	**H**	**I**	**K**	**L**	**M**	**N**	**P**	**Q**	**R**	**S**	**T**	**V**	**W**	**Y**
4.9	1.0	8.5	1.7	14.0	11.6	1.9	2.5	0.9	4.4	5.0	2.6	3.7	1.0	4.2	7.0	3.5	4.3	3.5	13.7

Supplementary Table S5. The percentage of amino acid composition at each position in LCDRs of the constructed library.

Position	A	C	D	E	F	G	H	I	K	L	M	N	P	Q	R	S	T	V	W	Y
**L24**	0.01	0.00	0.00	0.12	0.00	0.65	0.02	0.02	23.25	0.04	0.10	0.04	0.01	5.57	69.13	0.10	0.46	0.01	0.46	0.00
**L25**	64.08	0.04	0.01	0.01	0.02	0.14	0.00	0.01	0.00	0.01	0.04	0.00	0.38	0.01	0.00	33.85	0.99	0.41	0.00	0.01
**L26**	0.03	0.07	0.03	0.00	0.00	0.84	0.01	0.04	0.01	0.01	0.00	0.56	0.01	0.00	0.31	97.29	0.78	0.00	0.00	0.00
**L27**	0.00	0.00	0.04	1.78	0.00	0.03	1.60	0.00	0.08	0.51	0.00	0.00	0.15	94.47	1.26	0.01	0.00	0.01	0.00	0.04
**L27a**	0.12	0.05	0.13	0.00	0.07	0.80	0.03	0.44	0.04	0.01	0.01	2.81	0.12	0.00	1.32	88.72	5.13	0.03	0.02	0.15
**L27b**	0.14	0.00	0.01	0.00	0.14	0.02	0.01	5.62	0.00	46.89	0.00	0.00	0.12	0.00	0.01	0.00	0.01	47.00	0.00	0.00
**L27c**	0.39	0.01	0.00	0.27	4.17	0.06	0.05	0.61	0.02	77.31	0.10	0.00	0.04	0.20	0.17	0.67	0.06	15.78	0.01	0.08
**L27d**	0.28	0.10	3.07	0.07	3.11	0.16	27.79	0.01	0.61	0.51	0.00	2.65	0.10	0.73	0.51	4.89	0.06	0.04	0.28	55.03
**L27e**	0.30	0.05	0.51	0.25	0.11	1.76	0.15	0.71	0.23	0.07	0.05	4.61	0.25	0.01	5.32	80.11	5.18	0.12	0.01	0.22
**L27f**	2.12	0.03	1.44	0.09	1.33	0.27	0.03	0.08	0.02	0.19	0.00	0.16	3.92	0.02	0.03	88.08	0.60	0.41	0.00	1.16
**L28**	1.05	0.02	19.52	0.14	0.46	15.05	0.41	1.71	0.52	0.68	0.02	23.63	0.12	0.01	0.48	21.25	2.38	12.16	0.00	0.40
**L29**	0.44	0.02	0.58	0.11	0.35	14.47	0.09	38.36	0.84	0.77	0.14	18.55	0.11	0.02	0.81	10.77	1.42	11.98	0.00	0.16
**L30**	1.03	0.04	3.29	0.38	0.27	4.76	0.79	0.66	20.05	0.22	0.18	11.06	0.08	0.37	5.87	42.93	2.60	0.45	0.13	4.86
**L31**	0.46	0.02	4.44	0.13	0.14	1.41	0.64	1.35	1.58	0.05	0.06	40.45	0.12	0.06	2.91	29.75	15.54	0.15	0.02	0.72
**L32**	1.59	0.61	4.85	0.15	4.62	0.18	2.54	0.03	0.42	0.50	0.01	6.98	0.03	0.30	0.36	4.72	0.20	0.04	6.59	65.30
**L33**	0.01	0.00	0.00	0.00	1.01	0.00	0.00	0.64	0.00	94.41	1.52	0.00	0.00	0.00	0.00	0.10	0.01	2.28	0.01	0.01
**L34**	62.38	0.04	5.39	0.12	0.23	3.99	1.02	0.26	0.05	0.07	0.01	18.27	0.06	0.02	0.03	3.22	1.66	1.21	0.00	1.96
**L50**	19.17	0.06	15.94	5.40	0.18	20.53	0.29	0.05	6.96	5.06	0.23	0.23	0.11	0.67	1.04	1.62	1.74	0.64	19.56	0.54
**L51**	80.09	0.00	0.04	0.13	0.03	5.07	0.01	1.13	0.02	0.39	0.02	0.03	0.25	0.00	0.02	0.98	3.23	8.55	0.00	0.00
**L52**	0.50	0.03	0.02	0.00	1.08	0.02	0.02	0.09	0.00	0.08	0.00	0.05	0.23	0.00	0.01	94.90	2.58	0.05	0.01	0.33
**L53**	0.77	0.01	0.73	0.05	0.15	0.39	0.63	2.23	1.81	0.07	0.07	23.93	0.04	0.29	2.25	25.54	40.13	0.24	0.01	0.66
**L54**	0.00	0.01	0.00	0.00	0.24	0.13	0.01	0.02	0.06	37.68	0.08	0.01	0.05	0.02	60.40	0.41	0.09	0.28	0.51	0.00
**L55**	32.87	0.00	5.38	27.45	3.54	0.59	1.17	0.12	0.99	0.47	0.01	0.08	0.39	23.71	0.48	0.10	0.11	2.39	0.01	0.15
**L56**	1.74	0.06	0.16	0.03	0.41	1.00	0.03	0.46	0.07	0.07	0.05	0.93	3.07	0.00	0.83	60.40	30.45	0.11	0.00	0.12
**L89**	0.02	0.05	0.00	0.12	0.03	0.02	3.07	0.10	0.19	5.34	13.19	0.13	0.06	77.04	0.16	0.17	0.06	0.21	0.00	0.04
**L90**	0.01	0.01	0.03	0.64	0.00	0.02	4.46	0.01	1.52	0.44	0.01	0.34	0.08	91.17	0.71	0.18	0.06	0.08	0.02	0.23
**L91**	7.31	0.69	0.97	0.05	2.88	5.75	4.75	0.38	0.01	4.38	0.08	0.65	0.12	0.09	5.65	10.58	2.24	0.65	0.02	52.73
**L92**	1.28	0.21	9.53	0.90	1.67	12.88	2.58	2.80	2.00	5.49	0.11	16.85	0.02	0.28	1.06	6.85	6.00	0.86	0.09	28.54
**L93**	0.97	0.04	4.19	1.00	0.29	3.39	5.61	1.69	1.04	0.66	0.10	17.49	0.14	7.47	2.63	42.49	9.40	0.45	0.05	0.90
**L94**	3.47	0.08	0.48	0.05	8.01	0.57	0.47	2.64	0.08	7.84	0.30	1.18	1.76	0.09	0.53	18.03	23.26	1.53	16.21	13.42
**L95**	0.52	0.00	0.03	0.08	0.41	0.23	0.29	0.12	0.03	4.13	0.10	0.02	88.41	0.49	1.33	2.72	0.48	0.42	0.12	0.05
**L95a**	3.71	0.29	0.18	0.81	0.80	3.52	1.15	0.85	0.34	9.20	1.52	0.06	57.68	3.01	6.86	7.32	1.30	0.87	0.18	0.35
**L95b**	7.60	0.05	0.81	10.27	0.87	20.33	0.05	3.02	1.24	8.76	7.69	0.51	5.15	0.90	10.40	10.02	6.35	4.47	1.29	0.22
**L95c**	1.48	0.00	0.98	2.95	1.62	24.84	0.06	19.50	0.19	4.21	3.65	0.36	18.19	0.08	1.07	11.25	7.65	1.53	0.14	0.25
**L95d**	6.90	0.04	44.57	0.76	0.09	4.45	0.04	0.22	0.13	4.14	0.71	0.09	9.71	0.00	0.22	24.80	0.49	2.27	0.00	0.36
**L95e**	0.36	0.00	3.21	0.00	0.36	17.86	0.00	0.00	0.00	0.71	10.36	0.00	52.50	1.79	0.36	10.36	1.43	0.71	0.00	0.00
**L95f**	0.00	0.00	15.69	9.80	7.84	3.92	0.00	0.00	0.00	0.00	41.18	0.00	9.80	0.00	0.00	3.92	7.84	0.00	0.00	0.00
**L96**	0.44	0.32	0.36	0.21	12.67	1.36	1.33	20.60	0.19	27.82	0.20	0.61	8.12	1.15	5.01	1.21	0.56	4.45	3.84	9.58
**L97**	1.46	0.01	0.06	0.01	0.20	0.29	0.10	0.52	0.06	0.67	0.04	0.26	0.48	0.01	0.18	2.30	93.14	0.21	0.00	0.00

The percentages of the amino acid composition at each position in LCDRs having cut-off ≥ 0.01 are represented. In case of the amino acid compositions cut-off < 0.01 were expressed as 0.00.

Supplementary Table S6. The average percentage of amino acid composition in LCDR3 of the constructed library.

Amino acid composition (%)																			
**A**	**C**	**D**	**E**	**F**	**G**	**H**	**I**	**K**	**L**	**M**	**N**	**P**	**Q**	**R**	**S**	**T**	**V**	**W**	**Y**
2.4	0.1	5.4	1.8	2.5	6.6	1.6	3.5	0.5	5.6	5.3	2.6	16.8	12.2	2.4	10.1	10.7	1.2	1.5	7.1

Supplementary Table S7. Primers used for the generation of scFv sequences.

Primer name	5′ to 3′ Sequence
RSC-F	GAGGAGGAGGAGGAGGAGGCGGGGCCCAGGCGGCCGAGCTC
RSC-F_2	GAGGAGGAGGAGGAGGAGGCGGGGCCCAGGCGGCC
RSC-B	GAGGAGGAGGAGGAGGAGCCTGGCCGGCCTGGCCACTAGTG
